# Estrogen and estrogen receptors in kidney diseases

**DOI:** 10.1080/0886022X.2021.1901739

**Published:** 2021-03-30

**Authors:** Hao-Yang Ma, Shuang Chen, Yang Du

**Affiliations:** a Department of Geriatrics, Second Affiliated Hospital of Zhejiang University, Hangzhou, China; b Jiangsu Key Laboratory of Pediatrics, Nanjing Medical University, Nanjing, China; c Nanjing Key Laboratory of Pediatrics, Children’s Hospital of Nanjing Medical University, Nanjing, China

**Keywords:** Estrogen, estrogen receptors (ERs), kidney diseases, acute kidney injury (AKI), chronic kidney disease (CKD)

## Abstract

Acute kidney injury (AKI) and chronic kidney disease (CKD) are posing great threats to global health within this century. Studies have suggested that estrogen and estrogen receptors (ERs) play important roles in many physiological processes in the kidney. For instance, they are crucial in maintaining mitochondrial homeostasis and modulating endothelin-1 (ET-1) system in the kidney. Estrogen takes part in the kidney repair and regeneration *via* its receptors. Estrogen also participates in the regulation of phosphorus homeostasis *via* its receptors in the proximal tubule. The ERα polymorphisms have been associated with the susceptibilities and outcomes of several renal diseases. As a consequence, the altered or dysregulated estrogen/ERs signaling pathways may contribute to a variety of kidney diseases, including various causes-induced AKI, diabetic kidney disease (DKD), lupus nephritis (LN), IgA nephropathy (IgAN), CKD complications, etc. Experimental and clinical studies have shown that targeting estrogen/ERs signaling pathways might have protective effects against certain renal disorders. However, many unsolved problems still exist in knowledge regarding the roles of estrogen and ERs in distinct kidney diseases. Further research is needed to shed light on this area and to enable the discovery of pathway-specific therapies for kidney diseases.

## Introduction

Although estrogen is classically regarded as a reproductive hormone in mammalian species, it also plays an important role in many other physiological processes (e.g., cell growth, development and differentiation, lipid and glucose homeostasis, renal endocrine function, immune function, etc.) [1–5]. The effects of estrogen in physiological and pathophysiological conditions are mediated by two different structural receptor classes, namely, estrogen receptor (ER) α/β and G protein-coupled estrogen receptor (GPER) [6]. Estrogen and its receptors are implicated in the development and progression of various diseases, including cancer, osteoporosis, endometriosis, neurodegenerative disorders, as well as cardiovascular, metabolic, and autoimmune diseases [7–12].

Acute kidney injury (AKI) and chronic kidney disease (CKD) are posing substantial threats to global health. The interdependent relationship between AKI and CKD further adds complexity to the clinical picture [13]. Recent experimental and clinical studies have suggested that estrogen and ERs serve pathophysiological roles in kidney diseases, including AKI, diabetic kidney disease, lupus nephritis, IgA nephropathy, complications of CKD, etc. This review mainly examines the roles of estrogen and its receptors in certain kidney diseases. We also discuss the structures, functions, mechanisms, and modulation of ERs, providing the basis for potential therapeutic interventions.

## Structures and functions of ERs

As members of the nuclear receptor family, ERs are found mainly in the nucleus, but also in the cytoplasm and mitochondria [14]. The classical ER subfamily mainly consists of ERα and ERβ [15]. Both of them consist of six functional domains A–F. The NH_2_-terminal A/B domain contains a ligand-independent transactivation function-1 (AF-1). It’s noteworthy that the transactivation potency of AF-1 varies in a ligand-, cell type-, and promoter-specific manner [3]. The C domain (DNA binding domain) binds to DNA motifs called estrogen response elements (EREs). It also plays a part, to a minor extent, in the stability of ER dimerization [3]. The D domain is a hinge region between the C and E domains. It is involved in ER conformational changes, interaction with other transcription factors, nuclear translocation, and posttranslational modifications [16–18]. The E domain is identified as the ligand-binding domain (LBD) and the principal dimerization interface of the receptor, which contains a ligand-dependent activation function AF-2 [19,20]. The F domain is the least conserved region with high variability, and many nuclear receptors do not contain such a region [3]. ERα and ERβ share a high degree of conservation in their C and E domains, while the other domains are more divergent [21]. The synergistic effect of AF1 and AF2 is required in the transcriptional regulation mediated by both receptors [22,23]. However, it is still unclear how AF-1 and AF-2 activities are regulated cooperatively by ligands. Recent studies have shown that the AF-2 contains an AF-1 suppression function element and that AF-1 is regulated in an AF-2-dependent manner [24,25].

### ERα

In humans, ERα is encoded by the gene ESR1, located on chromosome 6, locus 6q25.1 [26]. ERα is primarily expressed in sex organs (breast, uterus and ovary, testes, epididymis, prostatic stroma), bone, liver, adipose tissue, cardiovascular, and central nervous system (CNS) [27,28]. The classic full-length 67 kDa ERα includes a DNA-binding domain, a ligand-binding domain, and two transcriptional activation functions (AF-1 and AF-2) [3]. In addition, two shorter isoforms (46 kDa ERα46 and 36 kDa ERα36) have been identified. ERα46 lacks the N-terminal region harboring AF-1, whereas ERα36 lacks both AF-1 and AF-2 and encodes a unique 29 amino acid sequence [29,30].

The functional role of ERα was first discovered from a clinical situation where a man bearing a mutation in the ERα gene developed a premature and severe metabolic syndrome [31]. It is now recognized that ERα is a key regulator of energy homeostasis and glucose metabolism and that the ERα pathway might represent a potential therapeutic target for the prevention or treatment of insulin resistance, type 2 diabetes mellitus, and non-alcoholic fatty liver diseases [16,32]. On the other hand, ERα is linked with a variety of cancers and metastases, including breast cancer, cervical cancer, lung carcinoma, and prostate cancer [33–36].

### ERβ

In humans, ERβ is encoded by the gene ESR2, located on chromosome 14 (14q23–24), and has five isoforms (ERβ1-5) [37]. These five isoforms exist as a result of alternative splicing of the last coding exon (exon 8). It is noteworthy that ERβ1 is the only full-function isoform with the native LBD and that the other isoforms do not have innate activities in their homodimeric forms but can heterodimerize with ERβ1 and enhance ERβ1-induced transactivation in a ligand-dependent manner [38,39]. ERβ and its isoforms have wider tissue distribution than ERα and they are expressed primarily during embryonic development and in the prostatic epithelium, bladder, ovary, colon, lung, adipose tissue, immune system, cardiovascular system, and CNS [27,40,41].

Similar to ERα, ERβ is involved in cellular differentiation, mitochondrial bioenergetics, lipid and glucose metabolisms, energy expenditure, etc. [42–44]. ERβ is generally thought to be a tumor suppressor gene and its expression is dysregulated in different cancers [45–47]. There is an increasing awareness that selective targeting of ERβ signaling pathways might be useful in the treatment of several inflammatory and proliferative diseases [48,49]. Studies support the idea that ERβ agonists can reasonably be used in hormone replacement therapy, early stage prostate and colon cancers, suppression of the immune system without negative effects on bone, and neuroprotection [50].

### GPER

In the last decades, studies have suggested that apart from the classical steroid receptors ERα and ERβ, the G protein-coupled estrogen receptor (GPER, formerly known as GPR30) also mediates the effect of estrogen in a rapid signaling pathway [51–53]. As a member of the G-protein coupled receptor superfamily, GPER is localized predominately within intracellular membranes in most cell types [54]. It is widely expressed in numerous tissues and organs, including the vessels, skeletal muscle, brain, heart, kidney, pancreas, and reproductive organs [55,56]. Studies have shown that GPER is involved in many physiological responses, including maintenance of vascular tonicity and blood pressure, reproduction, lipid and glucose metabolisms, immune and inflammatory responses, etc. [57–60]. For instance, mice lacking GPER exhibited metabolic syndrome, such as obesity, impaired glucose tolerance, or dyslipidemia [59,61]. Pharmacological modulation of GPER could promote pancreatic cell survival and improve glucose tolerance [62,63]. The effects of GPER are mediated *via* multiple signaling pathways, including the activation of adenylyl cyclase (AC)/protein kinase A (PKA), epidermal growth factor receptor (EGFR), PI3 kinases, as well as extracellular signal-regulated kinase (ERK) pathways and G protein-coupled pathways [64].

## Mechanisms of estrogen action

**Figure 1.**
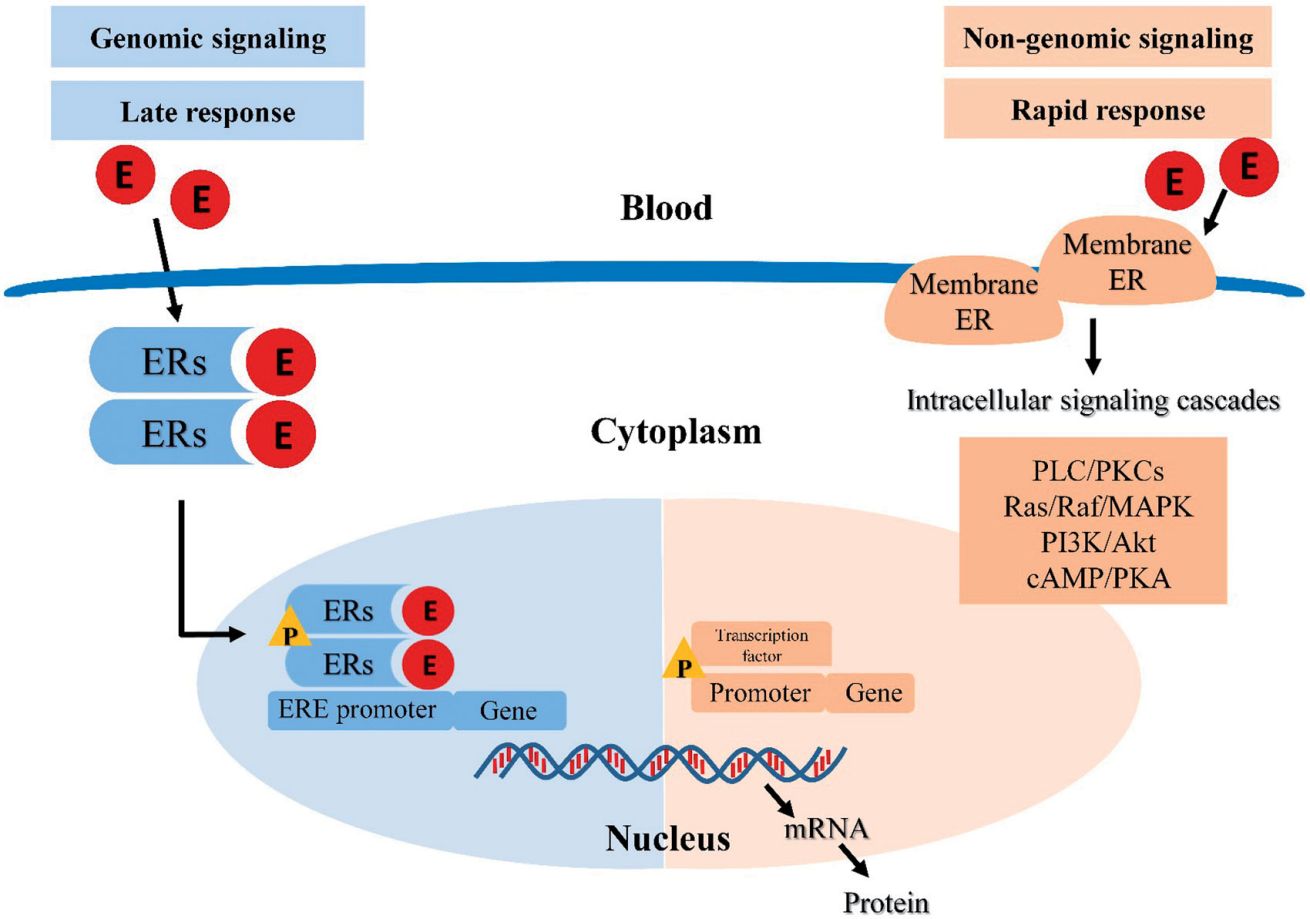
Classical (genomic) and non-genomic estrogen signaling pathways. E: estrogen; ERs: estrogen receptors; P: phosphorylation; ERE: estrogen receptor elements.

The mechanisms of estrogen action are categorized into classical (genomic) and rapid (non-genomic) ones. In the classical pathway, estrogen binds to the ERs in the cytoplasm, leading to ER dimerization and translocation to the nucleus, where the estrogen–ER complex interacts with ERE sequences in target genes [16]. This process typically occurs within hours [65]. In recent decades, however, rapid or ‘non-genomic’ effects of estrogen (also termed non-nuclear or membrane initiated steroid signaling) has been reported [66]. This occurs through the ER located in or adjacent to the plasma membrane, or through other non-ER plasma membrane-associated estrogen-binding proteins, which usually takes seconds or minutes [67]. GPER has been identified as one of the main estrogen-sensitive receptors responsible for the rapid non-genomic action of estrogen [68]. The classical (genomic) and non-genomic estrogen signaling pathways are illustrated in Figure 1 [22,69].

## Modulation of ERs

Selective estrogen receptor modulators (SERMs)  are antiestrogens designed to compete with estrogen and modulate ER activity in a tissue-specific manner [70,71]. For instance, tamoxifen can exhibit antagonistic effect on mammary tissue, whereas it can have agonistic effects on other tissues such as the uterus, bone, and heart [72]. Raloxifene acts as an estrogen agonist in bone and an estrogen antagonist in uterine and breast tissues [73]. Similarly, bazedoxifene functions as a pure antagonist in the breast and an agonist in the bone [74]. Since ERs are nuclear transcription factors involved in the regulation of a variety of physiological and pathological processes in humans, modulation of the receptors either by SERMs or by agonists/antagonists might be beneficial for the prevention and treatment of various diseases [27].

## Estrogen and ERs in kidney diseases

### Acute kidney injury (AKI)

#### Gender differences in AKI epidemiology

The incidence of AKI has been steadily increasing, particularly among elderly hospitalized patients [75]. Epidemiological studies suggested that the mortality rates of AKI patients in hospital settings (including intensive care units) ranged from 17.5% to 64.7% [75,76]. As a broad clinical syndrome encompassing different etiologies, AKI is characterized by an abrupt decline of glomerular filtration rate, which is associated with high morbidity and mortality [77]. Various etiologies include pre-renal azotemia, acute tubular necrosis, acute glomerular/interstitial nephritis, acute vasculitic renal diseases, acute post-renal obstructive nephropathy, and mixed forms [78].

Clinical studies have shown that sex disparities might influence the susceptibility, progression, and therapeutic response to AKI [79–81] and that female sex might have a protective effect on the development of AKI [79,82]. Though a recent retrospective cohort study of AKI complicating acute myocardial infarction-related cardiogenic shock suggested that female sex was independently associated with higher in-hospital mortality, it did mention that women with AKI were older (74 ± 12 years), which highlighted the potential role of estrogen in AKI [83]. Another interesting phenomenon is that compared with females, males were endowed with lower mitochondrial respiratory capacity and poor antioxidant defense system, exhibiting fragmented and smaller mitochondria [84,85]. Given that the disrupted mitochondrial homeostasis plays a prominent role in the pathogenesis of AKI [86], this might partially explain the sex disparities in AKI. The gender dimorphism was also observed in animal experiments where male mice/rats exhibited more severe AKI manifestations than their female counterparts through different mechanisms [87–91].

The role of silent mating type information regulator 2 homolog 1 (SIRT1) in mediating the renoprotective effects of estrogen on AKI has been systematically reviewed recently [92]. Experimental studies have indicated that SIRT1 exerts a protective effect against AKI through regulating oxidative stress, mitochondrial biogenesis, energy metabolism, inflammation and apoptosis [93–97]. The functional interaction between estrogen/ER and SIRT1 has been investigated in different disease models. Estrogen exerts protective effects against inflammation and mitochondrial dysfunction *via* ERα/SIRT1 pathway [98,99]. SIRT1 acts as an ERα co-activator and is required for modulation of ERα-signaling pathways [100]. In this regard, it is proposed that estrogen through SIRT1 might protect against AKI [92].

#### Estrogen and ERs in AKI settings

##### Renal ischemia–reperfusion injury (IRI)

Renal IRI is characterized by a temporary shortage and subsequent restoration of blood supply and oxygen delivery to the kidney, initiating a cascade of deleterious cellular responses leading to ROS generation, inflammation and tubular cell death, leading to AKI [101,102]. As one of the leading causes of AKI, renal IRI often occurs with kidney transplantation, postoperative hypotension, traumatic hemorrhage, cardiovascular surgery, cardiac arrest, and cardiopulmonary resuscitation [103–108].

Clinical studies regarding the association of gender with kidney allograft outcome have mixed results. While some clinical observations suggested that female recipients generally had a better graft function and survival than male counterparts, others indicated that the risk of developing graft failure varied in women stratified by age and donor sex [109–112]. Although the effect of gender disparity in the susceptibility and pathogenesis of IRI in the transplanted kidney is less well established in humans, the majority of experimental studies indicate that females had better outcome of kidney transplantation than males due to increased IRI tolerance [113]. This sex-related differences in IRI-induced AKI might be attributed to the depression of renal sympathetic nervous system with endogenous estrogen and the subsequent reduced regional noradrenaline level [89,114,115]. As expected, the supplementation of estrogen prior to the induction of IRI protected kidney function in female mice and neutered males. Studies also revealed that estrogen administration after cardiac arrest and resuscitation ameliorated AKI in both male and female mice [116,117]. In contrast, testosterone enhances kidney susceptibility to IRI through inhibiting the activation of nitric oxide (NO) synthases and Akt signaling [118]. Notably, renal IRI was exacerbated in female ERα knockout mice [113].

The roles of estrogen and ERs in the signaling mechanisms of IRI-induced AKI have been reported. Estrogen attenuated renal IRI through the activation of peroxisome proliferator-activated receptor γ (PPARγ), a nuclear receptor which plays a vital role in the maintenance of renal metabolic homeostasis [119–121]. The overproduction of renal endothelin-1 (ET-1), which plays a critical role in the initiation of AKI and the subsequent transition to CKD through its two receptors ET_A_ and ET_B_, was suppressed with the administration of estrogen in rats challenged with IRI [122–125]. This effect was probably mediated by ERβ and GPER1 in the kidney [126,127]. Estrogen also reduced inflammation and accelerated injured tubular cell regeneration in male rats after IRI-induced AKI [128]. In the uninephrectomized rat model of IRI, the renoprotective effect of estrogen was mediated by the activation of PI3K/Akt pathway followed by increased endothelial nitric oxide synthase (eNOS) phosphorylation in the affected kidneys [129]. Another mechanism might be estrogen-mediated antagonism on *N*-methyl-d-aspartate receptors (NMDAR) that were expressed in nephrons [130]. The activation of GPER1 also protected the kidney from IRI through enhancing glomerular endothelial barrier function and regulating the content of NO in renal interlobular artery smooth muscle and endothelial cells [131,132]. Renal Na^+^/K^+^-ATPase is a well-recognized energy-dependent sodium pump which helps maintain the electrolyte and fluid balance *via* manipulating the transport of certain solutes [133,134]. Under the circumstance of renal IRI, the impaired activity of Na^+^/K^+^-ATPase contributed to abnormal hydroelectrolyte status [135,136]. A sex disparity in the alterations of this enzyme was observed following renal IRI, favoring the protective role of estrogen in the stability and modulation of Na^+^/K^+^-ATPase upon various renal insults [137,138].

Together, these results demonstrate that there exists a sex dimorphism in IRI-induced AKI. However, the discrepancy between clinical observations and experimental findings regarding this aspect remains to be investigated. Estrogen and ERs might have potential therapeutic implications in the treatment of renal IRI, but recent studies have pointed out that the renoprotective effect of exogenous estrogen becomes evident only when administered at a dose above the physiological level [139]. It has also been doubted that whether the sequelae of renal IRI (i.e., subsequent kidney fibrosis) could actually be brought to a halt by estrogen. Therefore, the therapeutic effect of estrogen and its analogues derived from the existing animal studies needs to be cautiously interpreted when applied to human patients.

##### Septic AKI

Sepsis-induced AKI is the leading cause of AKI in the intensive care unit (ICU) and accounts for nearly half of all AKI events, which is associated with increased mortality and morbidity [106,140]. A multicenter prospective study including 1177 ICU patients with sepsis in 24 European countries reported a 51.2% incidence of AKI with a mortality rate of 41.2% [141]. A multicenter retrospective study of [146,148]. Chinese hospitalized adults found AKI in 47.1% of sepsis cases [142].

Studies regarding the role of estrogen in septic AKI have conflicting results. Feng et al. found that serum estrogen levels were correlated with the severity of renal dysfunction and that increased estrogen levels predicted the development of newly onset AKI within a month [143]. A recent post-hoc analysis of patients with sepsis from the Randomized Evaluation of Normal versus Augmented Level renal replacement therapy (RENAL) trial suggested that female sex was associated with improved survival in septic patients with severe AKI [82]. Trentzsch et al. found that female patients had lower rates of sepsis and renal failure than males following traumatic injury and hemorrhagic shock [144]. Animal studies also demonstrated that estrogen and its selective modulator raloxifene had protective effects on renal function in ovariectomized rats with sepsis through the activation of autophagy [145,146]. Several preclinical studies indicated that the activation of ERs (mainly ERα and ERβ) could regulate inflammatory responses and control sepsis-induced multiorgan dysfunction [147–149].

The exact mechanisms responsible for the roles of estrogen and ERs in septic AKI remain to be elucidated. Further studies are needed to uncover the potential roles of estrogen and ERs in mediating the gender differences in septic AKI. Though a majority of experimental studies have demonstrated the salutary effects of estrogen and ER agonists on the outcome of sepsis-induced multiple organ failure including AKI, clinical investigations have not reached a consensus in this respect. The remaining gap between the bench and the bedside prompts us to take into consideration the different clinical study protocols. For example, the hormonal status or the level of estrogen of a patient at the time of sepsis may have an effect on the clinical outcome [150,151]. Studies providing information regarding hormonal status (oral contraceptives, menstrual cycle, and hormone replacement therapy) at the time of patient enrollment are encouraged. It is important to note that kidney is just one of the many involved organs in sepsis and that the overall effects of estrogen in other organs or systems need addressing. Also, fluid resuscitation and antibiotics are still the mainstay of treatment for patients with sepsis and, therefore, estrogen-related therapies may be developed for the best interests of the individual patient.

##### Drug-induced AKI

Drug-induced AKI accounts for 0.7% to 26% of cases with AKI among hospitalized patients [152–154]. Various drugs that are metabolized in the kidney and cleared *via* glomerular filtration and/or tubular secretion may induce AKI from a variety of mechanisms [155]. In the proximal tubules, apical transport of the aminoglycosides and basolateral transport of cisplatin increase the risk for AKI [156]. Acute interstitial nephritis is another form of drug-induced AKI, which typically develops from medications that incite an allergic reaction (e.g., antibiotics, proton pump inhibitors, non-steroidal anti-inflammatory drugs, etc.) [157–159].

Studies regarding the sex differences in the susceptibility to drug-induced AKI have conflicting results. While some suggested that females were more vulnerable to aminoglycoside- and cisplatin-induced nephrotoxicity, others found no such gender disparities [160–162]. At least in the field of drug-induced acute interstitial nephritis, epidemiological characteristics showed a female preponderance [159,163]. This might be attributed to gender differences in the expression of drug transporters on proximal renal tubules [164]. However, the issue of gender differences in the susceptibility to nephrotoxins is far more nuanced. There is actually no impact of gender on the protein levels of some key drug transporters in human kidney samples, as revealed by liquid chromatography-tandem mass spectrometry-based targeted proteomics [165,166]. On the other hand, sex differences in the expression of kidney genes responsible for the metabolism of certain drugs have been confirmed in experimental studies [164,167]. For example, females could better handle mercuric chloride-induced tubular injury because of their correspondingly increased renal expression of organic anion transporter 1/3 (OAT1/3) and multidrug resistance-associated protein 2, leading to higher excretion of mercury and less likelihood of AKI [168]. A sex-hormone dependent pattern of the expression of OAT2 was also observed in mouse and rat kidneys (i.e., OAT2 could be upregulated by estrogen and progesterone) [169].

The roles of estrogen and ERs in drug-induced AKI have been investigated. Preclinical studies demonstrated that young females exhibited decreased susceptibility to cisplatin-induced AKI, highlighting the potential role of estrogen [154,170,171]. Moreover, estrogen and GPER1 agonist G-1 had protective effects against human tubular epithelial cell injury induced by methotrexate [172]. In a rat model of heavy metal-induced AKI, tamoxifen prevented mercury-induced toxicity on mitochondrial energy-dependent functions in the kidney [173]. In contrast, fulvestrant, an ERα down-regulator, exacerbated kidney injury in rats with AKI induced by gentamicin [174,175]. Further studies are warranted to determine the roles of estrogen and ERs in drug-induced AKI.

It is well established that renal tubules are very susceptible to various insults including a range of commonly used drugs [176]. However, there exist distinct differences regarding the vulnerability to certain drugs among different segments of tubules or regions of the kidney. Whether or not estrogen or ERs confer comprehensive protection within the kidney is still unknown. Besides, drug-induced nephropathy can also be accompanied by other organ disorders such as hepatotoxicity and thrombocytopenia [177,178]. Management of these comorbidities, therefore, is equally important. A physician should have a high index of suspicion for the risk factors associated with drug-induced AKI in that the underlying health conditions of the patients may have a great impact on the clinical outcome. Correcting risk factors and treating underlying diseases should be emphasized.

### Chronic kidney disease (CKD)

#### Gender differences in CKD epidemiology

According to the 2017 KDIGO guidelines, CKD is defined as abnormalities of kidney structure or function, present for more than 3 months, with implications for health [179]. Major causes of CKD include diabetic nephropathy, IgA nephropathy, lupus nephritis, and membranous nephropathy [180,181].

Epidemiological studies suggested that females experienced slower renal function decline than males, possibly owing to the protective effect of estrogen or the damaging effect of testosterone [182,183]. Studies also revealed that premenopausal women (particularly those less than 45 years old) who underwent bilateral oophorectomy were at higher risk of developing CKD [184], highlighting the renoprotective effect of estrogen [185,186]. On the other hand, CKD itself is associated with hypothalamic–pituitary–ovarian dysfunction, which results in the earlier onset of menopause in uremic women [187]. The estrogen-based hormone replacement therapy seems to ameliorate renal dysfunction and delay CKD progression in postmenopausal patients, which might be partially due to the increased renal NO production and reduced oxidative stress [188–190]. Similarly, animal studies also indicated that the progression of kidney disease was slower in females.

#### Estrogen and ERs in CKD settings

##### Diabetic kidney disease (DKD)

As a major microvascular complication of diabetes mellitus, DKD is the most common cause of end-stage renal disease (ESRD) in developed countries. DKD has severe individual and societal consequences, owing to its high morbidity, mortality, and health-care costs [191]. Clinical studies regarding the sex-specific differences in the prevalence and progression of DKD are inconsistent. While some studies reported a higher incidence of DKD and subsequent ESRD in male population, others suggested a female predominance, or no such differences [192–194]. Different study protocols (e.g., age, race, types of diabetes, etc.) and confounding factors might explain this inconclusive link between sex and DKD [195]. The role of sex hormones including estrogen in the setting of DKD has not yet been determined. Nevertheless, a majority of studies have shown that an imbalance of sex hormones does exist among both male and female patients with diabetes [196,197]. On the other hand, animal studies supported the concept of renal protection in DKD with estrogen [198–200].

The roles of estrogen and ERs in DKD have been investigated. It was reported that the deficiency of estrogen (ovariectomy) could exacerbate renal pathological manifestations (e.g., glomerulosclerosis and tubulointerstitial fibrosis) in rats with diabetes induced by streptozotocin (STZ). The supplementation of estrogen or raloxifene attenuated these changes through reducing lipid peroxidation and oxidative stress [199,201,202]. Similarly, in male diabetic mice with or without orchiectomy, estrogen effectively inhibited DKD progression (e.g., reduced glomerulosclerosis, albuminuria and glomerular hyperfiltration) [198]. In a model of spontaneous DKD, the estrogen treatment attenuated mesangial expansion and glomerular basement membrane (GBM) thickening but failed to ameliorate proteinuria and glomerulosclerosis in male Otsuka-Long-Evans-Tokushima-Fatty rats [203]. Mechanistic studies showed that estrogen exerted its renoprotective effect on DKD through upregulating matrix metalloproteinase (MMP)-2 and MMP-9 to accelerate the degradation of extracellular matrix (ECM) [204]. In addition, estrogen attenuated albuminuria and ECM deposition by regulating the expression of transforming growth factor-β1 (TGF-β1) and its downstream signaling pathway [205]. Moreover, both estrogen and raloxifene ameliorated albuminuria and mesangial expansion in ovariectomized db/db mice, possibly *via* inhibiting TGF-β1-induced fibronectin transcription and activator protein-1 (AP-1) activity [206].

ERα gene polymorphisms have been associated with the risks of developing CKD with type 2 diabetes mellitus in the African American population and of developing DKD in girls with type 1 diabetes [207,208]. ERα and its splice variants exhibited protective effects against DKD induced by STZ in female mice, as evidenced by reduced glomerular size, hyperfiltration, macrophage infiltration, and proteinuria [209]. The ERα signaling was proposed as one of the renal signaling pathways involved in the pathogenesis of DKD and might be a promising target for the treatment of nephropathy in diabetic patients [210].

Since glomerular mesangial cell (MC) and podocyte injuries are involved in the progression of proteinuria and DKD, quite a few studies have focused on the effects of estrogen and ERs on modulating these cellular processes [211]. Both ERα and ERβ are expressed in human/murine MCs and podocytes [212,213]. It was demonstrated that estrogen played a protective role in the regulation of proliferation and apoptosis of these cells *via* its receptors. For example, estrogen could increase the degradation of ECM to slow the progression of DKD through upregulating MMP-9 in MCs [213]. It also inhibited podocyte apoptosis through binding to ERβ, which was associated with the activation of the JAK2/STAT3 signaling pathway [214]. Moreover, estrogen or tamoxifen could improve albumin excretion, reduce glomerular size, and decrease matrix accumulation *via* upregulating the expression of ERβ and downregulating TGF-β in podocytes from db/db mice [215]. Podocytes isolated from these mice had a higher level of F-actin and lower level of caspase-9, indicating that estrogen might protect against podocyte injury in DKD through regulating both actin cytoskeleton and apoptosis [216]. In ERα knockout mice, podocyte injury (increased desmin and decreased nephrin and Wilms tumor-1) and apoptosis developed, and the estrogen treatment could prevent these changes *via* the activation of extracellular signal-regulated protein kinase (ERK) signaling pathway [217,218].

Studies regarding the role of GPER1 in DKD are limited. GPER1 agonist icariin was reported to exert protective effects against oxidative stress and fibrosis in male rats with DKD induced by STZ [219]. GPER1 was crucial in regulating MC migration and ECM production in response to TGF-β1 [220]. It was through GPER1 that icariin reduced the deposition of type IV collagen and fibronectin induced by high glucose in human/rat MCs by inhibiting TGF-β/Smad and ERK1/2 signaling pathways [221]. The activation of GPER1 also inhibited high glucose-induced podocyte apoptosis by modulating Bcl-2 expression and mitochondrial translocation [222].

The altered renin–angiotensin system (RAS) plays a crucial role in the context of DKD. Male STZ-induced diabetic mice administered with angiotensin II had more prominent albuminuria, glomerular hypertrophy and mesangial expansion than females [223]. Female rats had lower levels of albuminuria and renal angiotensinogen (AOGEN) mRNA compared with male rats in the development of STZ-induced DKD [224]. This indicates the sex dimorphism regarding RAS in DKD, highlighting the potential role of estrogen in this disease.

In brief, a majority of experimental studies back up the idea that estrogen might play a protective role in DKD through attenuating glomerular MC and podocyte injuries *via* its receptors and through its sex dimorphism in RAS. However, many controversies and questions remain regarding the roles of sex hormones in the pathophysiology and progression of DKD. The imbalance of sex steroids (e.g., estrogen versus testosterone) has been confirmed in the setting of diabetes from clinical trials, which suggests that restoration of hormonal homeostasis is far more important than just supplying estrogen as is done in most of the animal studies. Diabetes mellitus, especially type 2, is recognized as the cardiovascular disease equivalent and is associated with other end-organ complications such as retinopathy and neuropathy. The potential role of estrogen or ERs in these aspects has yet to be investigated. Moreover, the complexity and diversity of signaling networks involved in diabetes require more drugs developed to specifically target these dysregulated pathways.

##### Lupus nephritis (LN)

As one of the most severe manifestations of systemic lupus erythematosus (SLE), LN is characterized by proteinuria, hematuria and progressive renal dysfunction. It affects over half of all patients diagnosed with SLE and is a major risk factor for overall morbidity and mortality [225]. The female to male incidence of SLE mounts to nine during the fourth decade of life and declines subsequently until the seventh or eighth decade, disproportionately affecting women of reproductive age [226].

The predominance of females among patients with SLE underlines a pathogenic role for female hormones including estrogen [227]. Though mixed results exist regarding whether exogenous estrogen (either oral contraceptives or hormone replacement therapy) may increase the risk for SLE, endogenous estrogen and ERs have been confirmed to be associated with the modulation of both innate and adaptive immune responses in SLE [228–230]. The development and function of immune cells such as T/B cells and plasmacytoid dendritic cells (DCs), as demonstrated by a large body of literature, can be directly influenced by estrogen [231,232]. For instance, estrogen can interfere with the normal tolerance of naive DNA-reactive B cells and amplify the activation of autoreactive B cells [233,234]. Clinical studies further revealed that a baseline sex-biased difference of differentially expressed genes (DEGs) in B cells existed between healthy females and males, and that some of the DEGs were relevant to estrogen-induced type I interferon (IFN)/B cell activator factor (BAFF) signaling pathway [235,236]. Moreover, estrogen could stimulate the expression of CD40 ligand, a molecule involved in the pathogenesis of LN, in peripheral T cells isolated from patients with SLE [232,237]. The detrimental impact of estrogen on SLE was also confirmed by its inhibitory effect on T cell apoptosis, thus allowing for the persistence of autoreactive cells [231]. One randomized controlled trial evaluating the efficacy of fulvestrant in SLE patients found that selective ERs blocking could reduce disease activity as revealed by decreased expression of T cell activation markers and improved SLE disease activity index (SLEDAI) [238]. Interestingly, studies conducted from different nations have shown that male SLE patients had more severe disease patterns and worse prognoses compared with females. For instance, renal involvement, especially type IV LN with nephrotic syndrome, was more frequent in males [239–247].

In murine models of lupus, female mice were more susceptible to the development of glomerulonephritis [248] and the estrogen treatment accelerated the progression of LN [249]. This sex discrepancy suggests a potential role for estrogen in this autoimmune disease. A majority of animal studies concluded that estrogen might play a detrimental role in the pathogenesis of LN. The estrogen treatment could induce a lupus phenotype with kidney damage in wild-type mice and resulted in more immune cells expressing ERα (e.g., CD4^+^ and CD8^+^ T cells, dendritic cells, and macrophages) in autoimmune-prone mice [250]. The administration of estrogen to lupus-prone mice increased the levels of BAFF as well as anti-C1q and anti-dsDNA antibodies, and expanded the population of self-reactive idiotypic B and T cells, thereby accelerating the progression of glomerulonephritis in lupus [249,251]. Through binding to ERs, environmental estrogens (e.g., diethylstilbestrol and bisphenol-A) that mimic estrogenic activity also enhanced autoantibody production and IgG immune complex deposition in the kidney in lupus-prone mice after orchiectomy [252].

Studies of ERs in the pathogenesis of LN and other immune-mediated glomerular diseases have mixed results. Those in favor of the detrimental role of ERs argued that ERα acted in a B cell-intrinsic manner to promote B cell activation specifically in female lupus-prone mice, and that its deletion in B cells attenuated autoantibody production and glomerular immune complexes [253]. Furthermore, ERα knockout female mice developed less severe nephritis induced by nephrotoxic serum while manifesting normal autoimmune humoral response [12]. Similarly, other studies indicated that ERα deficiency conferred protection against proteinuria and tubular injury in female lupus-prone mice [254,255]. However, Scott et al. pointed out that previously reported ‘ERα knockout mice’ were not in fact ERα null but expressed an N-terminally truncated ERα (ERα short, similar to an endogenously expressed ERα46 variant), and that these mice were protected from LN after ovariectomy only if supplemented with estrogen [256,257]. This suggested that the ERα short protein had a protective role in LN and that endogenous ERα variants might represent a potential therapeutic target. Likewise, Shim et al. suggested that mice lacking ERα rather than lacking ERβ developed autoimmune glomerulonephritis, proteinuria, and plasma cell infiltration in the kidney without antigen challenge [258]. Clinical data have shown that ERα polymorphism is associated with SLE susceptibility in the overall and Asian populations as well as the renal and cutaneous involvements [259–261].

Mechanistic studies regarding the roles of estrogen and ERs in LN and immune-mediated glomerular injury are limited. Estrogen was reported to enhance the activation of conventional DCs through modulating IFN-dependent and independent pathways in both wild-type and lupus-prone mice [262]. ERα was required for Toll like receptor (TLR)-induced inflammation and the generation of both plasmacytoid DCs and interleukin-17 (IL-17) producing cells, all of which were implicated in SLE [263,264]. Another possible mechanism was that it was through ERα that the estrogen induced the expression of tumor necrosis factor-like weak inducer of apoptosis (TWEAK) and aggravated LN [265].

In conclusion, epidemiological studies and the majority of animal studies highlight the possibility of the involvement of estrogen/ERs in the pathophysiology of LN. The specific mechanisms underlying its role require further investigation. A recent study revealed that aberrant posttranslational modification of steroid receptors including ERα in T cells contributed to the gender-bias of SLE and that targeting ERα could improve the symptoms of lupus patients [266]. Since there are limited clinical trials investigating the efficacy of ERα antagonism in these patients, this pioneering finding may provide a novel molecular basis for future precision treatment.

##### IgA nephropathy (IgAN)

IgAN is the most common glomerulonephritis globally leading to ESRD [267]. Mesangial hypercellularity with deposition of IgA-containing immune complexes is one of the key pathological features.

Male gender was an independent risk factor for developing ESRD in IgAN and male patients presented with worse clinicopathologic manifestations than females [268,269]. Consistently, an animal study suggested that trichothecene vomitoxin (VT)-induced IgAN had a male predilection in B6C3F1 mice and that these males had more severe disease patterns [270]. To further investigate the role of estrogen in IgAN, a study found that castration of female B6C3F1 mice increased the severity of VT-induced IgAN, but supplementation with estrogen did not attenuate this effect but rather increased disease severity [271].

The polymorphism of ERα gene might be associated with the pathogenesis of IgAN [272]. Among several pathways regulating the proliferation of MCs in IgAN, ERα acted as a hub protein that could affect a set of proteins and transcription factors involved in the disease process [273]. A recent study using bioinformatics analysis based on the Gene Expression Omnibus database found that some of the key genes upregulated in IgAN were linked with the estrogen signaling pathway [274]. Another study reported that the expression of glomerular ERα in IgAN renal tissue decreased with the increasing severity of disease, proposing ERα as an independent factor affecting the prognosis of patients with IgAN [275]. Further studies are needed to elucidate the roles of estrogen and its receptors in the pathogenesis of IgAN.

##### Other CKD models

In animal models of CKD, estrogen was reported to exert protective effects against renal pathologies (e.g., glomerular hypertrophy, atrophic tubules, loss of podocytes, etc.) through attenuating oxidative stress and inflammation [276–278]. In the aging Dahl salt-sensitive (DSS) rats with ovariectomy, estrogen could attenuate glomerulosclerosis and tubulointerstitial fibrosis [279]. Spontaneously hypertensive stroke-prone (SHRSP) rats with uninephrectomy treated with estrogen had reduced albuminuria, glomerulosclerosis, and tubulointerstitial fibrosis [276]. Estrogen also preserved the contralateral kidney function in rats with unilateral ureteral obstruction (UUO), as revealed by reduced expressions of TGF-β and α-smooth muscle actin (α-SMA) [280]. As a SERM, tamoxifen conferred protection against kidney fibrosis induced by UUO *via* modulation of ERα-mediated TGF-β1/Smad pathway [281]. Its anti-fibrotic effect was also confirmed in a model of chronic nephropathy (hypertensive nephrosclerosis by chronic inhibition of NO synthesis) where treated rats had less albuminuria, glomerulosclerosis and interstitial fibrosis than untreated ones [282].

The role of ERs has also been investigated. In an adenine-induced CKD model, male rats developed more severe kidney damage than female ones, which might be associated with decreased renal expression of ERα [283]. However, a study using cotton rats to simulate the spontaneous CKD in the elderly population showed that both ERα and ERβ were strongly present in the renal tubules, which correlated with renal cystic lesions, inflammation and fibrosis [284]. Renal tubular epithelial cell regeneration and proliferation contribute to chronic kidney fibrosis, which ultimately leads to CKD [285,286]. Estrogen participated in the renal tubular regeneration process by modulating cell proliferation through the activation of both ERα and GPER1 receptor [287]. The role of ERα in kidney regeneration and growth was further confirmed in an animal model, where ERα knockout female mice exhibited reduced compensatory kidney growth [288].

Taken together, a majority of the studies suggest that estrogen might exert protective effects against CKD through ameliorating renal fibrosis and that SERMs might be a group of drugs of interest for CKD. However, pharmacokinetics in CKD should be taken into account because of the altered renal and non-renal drug clearance in this population [289]. SERMs such as tamoxifen and raloxifene have been confirmed to be associated with the risks of deep vein thrombosis and pulmonary embolism [290,291]. The long-term safety and efficacy of these agents need to be investigated. Besides, little progress has been made regarding the prevention and reversal of kidney fibrosis up to now. Whether these drugs confer protection to CKD patients is still unclear.

### Complications of CKD

#### Chronic kidney disease-mineral and bone disorder (CKD-MBD)

CKD-MBD is a major complication of CKD characterized by biochemical abnormalities, bone disorders, and vascular/soft tissue calcification, contributing to cardiovascular disease and mortality [292]. The abnormal metabolisms of calcium, phosphorus, parathyroid hormone (PTH), and vitamin D are key disturbances in CKD-MBD [293].

Physiologically, estrogen takes part in the regulation of phosphorus homeostasis by activating ERα/ERβ-mediated phosphate transporter NaPi-IIa in kidney proximal tubules [294]. Studies regarding the impact of estrogen on phosphate metabolism are mainly confined to the general population. The endogenous estrogen levels in older men were inversely associated with serum phosphorus levels [295]. Consistently, postmenopausal women receiving estrogen treatments had lower serum levels of phosphorus by increasing the urinary excretion of phosphorus [296]. Another SERM, bazedoxifene, could reduce serum phosphorus levels, thereby improving renal functions in postmenopausal osteoporotic women without severe renal insufficiency [297].

CKD-associated osteoporosis is more prominent in postmenopausal uremic patients, highlighting the potential role of estrogen in the disease process [298,299]. For instance, raloxifene was effective in improving bone mineral density (BMD) in postmenopausal osteoporotic women with CKD [300–303]. Further studies revealed that ERα gene polymorphism might dictate the different outcomes of BMD in postmenopausal hemodialysis patients who received raloxifene [304]. Animal studies showed that the deficiency of estrogen (ovariectomy) contributed to the impaired fixation of titanium implants in the femurs and further bone loss in the mandibles of uremic mice (5/6 nephrectomy) [305,306]. In an animal model of progressive CKD (male Cy/+ rats with autosomal dominant cystic kidney disease), raloxifene improved skeletal material properties (those independent of bone mass) and structural properties [307]. Mechanistic studies revealed that estrogen could inhibit PTH-stimulated osteoclast-like cell formation through blocking both the cAMP-dependent protein kinase (PKA) and the calcium/PKC pathway [308,309].

Vascular calcification is highly prevalent in the CKD population, which is associated with major adverse cardiovascular events. Hyperphosphatemia and the abnormality of renin–angiotensin–aldosterone system are contributing factors for this phenomenon [310,311]. Studies focusing on the effects of estrogen on phosphate/angiotensin II-induced vascular smooth muscle cells (VSMCs) proliferation revealed that estrogen and raloxifene prevented the mineralization of VSMCs [312]. Further analysis revealed that this protective effect was mediated by the transactivation of growth arrest-specific gene 6 (Gas6) and upregulation of liver kinase B1 (LKB1) in the presence of ERα [313,314]. A randomized controlled trial suggested that estrogen, which had a favorable effect on oxidative stress, might protect against atherosclerotic cardiovascular disease in postmenopausal women with ESRD on hemodialysis [315].

In conclusion, these results suggest that estrogen and SERMs are of clinical significance in the management of CKD-MBD through attenuating biochemical abnormalities, bone disorders, and vascular calcification. Potential adverse effects of these agents are still to be alerted, especially in hemodialysis patients. Although there were no reported breast cancer or thromboembolism associated with the use of raloxifene by the aforementioned studies, the long-term safety and efficacy of SERMs need to be confirmed by larger prospective studies with longer follow-ups. Moreover, the mainstays of therapy for CKD-MBD are dietary phosphorus restriction, modification of dialysis prescriptions, phosphate binders, calcitriol or vitamin D analogs, and calcimimetics [316]. Parathyroidectomy (PTX) is considered when patients develop drug resistance or side effects [317]. Recent studies also confirmed the feasibility and efficacy of microwave ablation treatment for patients ineligible for PTX [318,319]. Therefore, more studies are warranted to evaluate whether there is an additional benefit of adding estrogen or SERMs to the established treatment regimen.

#### Uremic coagulopathy

Epidemiological studies revealed an increased susceptibility to major bleeding events in dialysis patients [320,321]. Potential etiologies include platelet dysfunction, dysfunctional von Willebrand factor, decreased production of thromboxane, uremic toxins, and anemia [322,323]. The bleeding tendency in uremic patients was further complicated or exacerbated by their comorbidities and medications (e.g., hypertension, atrial fibrillation with warfarin therapy, cerebrovascular disease with anticoagulants) [318,324–326]. For patients who were refractory to routine interventions for uremic bleeding, conjugated estrogens might be another option [327–329]. Overall, there lacks concrete data regarding the efficacy of estrogen in the prevention and treatment of bleeding in CKD patients. Most of the studies investigating the hemostatic effect of estrogen were confined to the general population, particularly in the setting of recurrent bleeding from arteriovenous malformations, and still there were conflicting results [330,331]. Several case reports have suggested that estrogen, as part of the hormonal therapy, might be effective in the management of recurrent bleeding or hematoma in uremic patients [332–334]. An animal study suggested that estrogen could shorten the prolonged bleeding time in uremic rats and that this hemostatic effect was neutralized by ER antagonists [335]. A possible hypothesis was that the impaired l-arginine-NO signaling under uremic conditions led to acute endogenous deprivation of estrogen and resulted in inhibited platelet aggregation and adhesion [336–338].

The long-term effects of estrogen as a component of hormone therapy in postmenopausal women have been well described by the Women's Health Initiative trial and related studies, and a consensus has been reached that women who start hormone therapy within 10 years of menopause onset may have less risk of thromboembolic complications [339,340]. However, this notion should be taken prudently in CKD population, as suggested by KDOQI guidelines and several studies [341–343]. CKD is an established risk factor for venous thromboembolism [344,345]. Patients with CKD are at high risk for thromboembolic events due to endothelial dysfunction and retention of indolic solutes [346,347]. Estrogen can cause thrombosis through elevating the levels of prothrombotic factors and decreasing the levels of protein S and antithrombin [348]. Because of the altered drug pharmacokinetics in patients with CKD, exogenous estrogen treatment might be associated with an increased risk of arteriovenous access thrombosis [348–350]. Likewise, an animal study suggested that the estrogen supplementation in rats with renal impairments produced thrombotic microangiopathic lesions in the kidneys [351].

The dual effect of estrogen in uremic coagulopathy needs further investigation. Clinical application of estrogenic agents in the treatment of uremic bleeding requires more explorations. The risk–benefit ratio of estrogen in postmenopausal women with advanced CKD is still unknown and clinical trials addressing this issue are needed.

## Conclusions and research gaps

Beyond its conventional role in the reproductive system, estrogen also functions in diverse developmental and physiological processes through its different receptors. Estrogen and ERs are crucial in maintaining mitochondrial homeostasis and modulating ET-1 system in the kidney, which points forward to their indispensable roles in normal kidney function. In kidney proximal tubules, estrogen takes part in the regulation of phosphorus homeostasis *via* its receptors. Estrogen and modulation of ERs have been shown to exert anti-oxidative stress and anti-fibrosis effects in CKD models. On the other hand, studies have revealed the involvement of estrogen and ERs signaling pathways in some autoimmune kidney disorders such as LN.

In light of these findings, the altered or dysregulated estrogen/ERs signaling pathways contribute to a variety of diseases (Table 1). Moreover, given the fact that sex dimorphism exists in certain renal disease entities revealed by epidemiological studies, the roles of estrogen and ERs cannot be overemphasized in the pathogenesis and prognosis of kidney diseases. Indeed, clinical and experimental studies have shown that the level of estrogen or ERα gene polymorphism influences the susceptibilities or dictates the outcomes of several renal diseases. Based on the evidence provided in this review, we propose the concept that targeting estrogen/ERs signaling pathways, either by agonizing or by antagonizing, might works in patients with certain kidney diseases.

**Table 1.** Summary of key estrogen/ER signaling pathways and potential drugs in kidney diseases.

Kidney diseases	Animal/cell models	Key estrogen/ER signaling pathways	Potential drugs
AKI	IRI AKI after cardiac arrest and cardiopulmonary resuscitation Sepsis-associated AKI Mercury/cisplatin/gentamicin-induced AKI Methotrexate-induced tubular epithelial cell injury	Estrogen-renal sympathetic nervous system (↓)-regional noradrenaline (↓) Estrogen-PPARγ (+) Estrogen-ERβ/GPER-ET1 (↓) Estrogen-PI3K/Akt (+)-eNOS phosphorylation (↑) Estrogen-NMDAR (−) GPER-NO Estrogen-Na+/K+-ATPase Estrogen-OAT2 (↑)	Raloxifene Tamoxifen
DN	STZ-induced DN db/db mice Otsuka-Long-Evans-Tokushima-Fatty rats High glucose-induced mesangial cell/podocyte injuries	Estrogen-lipid peroxidation/oxidative stress (↓) Estrogen-MMP2/9 (+)-ECM (↓) Estrogen-TGFβ1 (−)-FN/AP-1 (−) Estrogen-ERK (+) ERβ-JAK2/STAT3 (+) GPER1-TGFβ/Smad (−)-ERK1/2 (−)	Raloxifene Tamoxifen Icariin
LN	MRL/lpr mice NZB/WF1 mice Chronic GVHD-induced LN	Estrogen-autoreactive B cells (↑)/T cell apoptosis (↓) Estrogen/ERα-BAFF (+) ERα-TLR (+) Estrogen-IFN dependent/independent pathways Estrogen/ERα-TWEAK	Fulvestrant
IgAN	VT-induced IgAN	ERα-MCs proliferation	
CKD	Aging DSS rats SHRSP rats with uninephrectomy UUO-induced kidney fibrosis Hypertensive nephrosclerosis induced by inhibition of NO synthesis Adenine-induced CKD	Estrogen-renal NO (↑)-oxidative stress (↓) Estrogen-TGFβ/α-SMA (−) ERα-TGFβ/Smad ERα/β-cystic lesions/inflammation/fibrosis Estrogen-ERα/GPER-kidney regeneration and growth	Tamoxifen
CKD-MBD	5/6 nephrectomy Male Cy/+ rats with autosomal dominant cystic kidney disease PTH-stimulated osteoclast-like cell formation Phosphate/Angiotensin II-induced VSMC proliferation	Estrogen-ERα/ERβ-NaPi-IIa (+) Estrogen-cAMP dependent PKA (−)-calcium/PKC (−) Estrogen/ERα-ERα-GAS6 (+)-LKB1 (↑)	Bazedoxifene Raloxifene
Uremic coagulopathy	Uremic rats	Impaired L-arginine-NO signaling → estrogen (↓) → platelet aggregation/adhesion (-) Estrogen-protein S/antithrombin (↓)	Conjugated estrogens

AKI: acute kidney injury; IRI: ischemia-reperfusion injury; PPAR: peroxisome proliferator-activated receptor; ET1: endothelin-1; eNOS: endothelial nitric oxide synthase; NMDAR: N-methyl-D-aspartate receptors; NO: nitric oxide; OAT2: organic anion transporter 2; DN: diabetic nephropathy; STZ: streptozotocin; MMP: matrix metalloproteinase; ECM: extracellular matrix; TGFβ: transforming growth factor β; FN: fibronectin; AP-1: activator protein-1; ERK: extracellular signal-regulated protein kinase; JAK2/STAT3: Janus kinase 2/signal transducer and activator of transcription 3; LN; lupus nephritis; GVHD: graft-versus-host disease; BAFF: B cell activating factor; TLR: toll-like receptor; IFN: interferon; TWEAK: tumor necrosis factor-like weak inducer of apoptosis; IgAN: IgA nephropathy; VT: trichothecene vomitoxin; MCs: mesangial cells; CKD: chronic kidney disease; DSS: Dahl salt-sensitive; SHRSP: spontaneously hypertensive stroke-prone; UUO: unilateral ureteral obstruction; α-SMA: α-smooth muscle actin; CKD-MBD: chronic kidney disease-mineral and bone disorder; PTH: parathyroid hormone; VSMC: vascular smooth muscle cell; cAMP: cyclic adenosine monophosphate; PKA: protein kinase A; PKC: protein kinase C; GAS6: growth arrest-specific gene 6; LKB1: liver kinase B1.

However, many gaps exist in knowledge regarding the roles of estrogen and ERs in distinct kidney diseases and further research is necessary to address these areas. The gender differences in the setting of AKI does not necessarily mean this is the case in every single cause for AKI. Likewise, the functional role of estrogen or ERs confined to one disease entity cannot readily be replicated in another and thus should be analyzed separately. Besides, evidence regarding the exact roles of estrogen and ERs is mixed, particularly in some autoimmune kidney disorders. Although modulation of ERs or supplementation of estrogen has renoprotective effects in several experimental models, there is still a long way to go before it can be applied to clinical trials. The benefits of SERMs have been witnessed in patients with CKD, however, adverse events and long-term outcomes should be assessed.

The therapeutic potential of targeting estrogen/ERs signaling pathways remains to be tested. Moreover, by no means does targeting these mechanisms fully tackle renal disorders, considering the complex signaling networks involved. Further experimental and clinical studies are warranted to comprehensively understand the roles of estrogen and ERs in different kidney diseases.

## References

[1] Barros RP, Gustafsson J. Estrogen receptors and the metabolic network. Cell Metab. 2011;14(3):289–299. 21907136 10.1016/j.cmet.2011.08.005

[2] Kitajima Y, Ono Y. Estrogens maintain skeletal muscle and satellite cell functions. J Endocrinol. 2016;229(3):267–275. 27048232 10.1530/JOE-15-0476

[3] Arnal J-F, Lenfant F, Metivier R, et al. Membrane and nuclear estrogen receptor alpha actions: from tissue specificity to medical implications. Physiol Rev. 2017;97(3):1045–1087. 28539435 10.1152/physrev.00024.2016

[4] El-Gendy AA, Elsaed WM, Abdallah HI. Potential role of estradiol in ovariectomy-induced derangement of renal endocrine functions. Ren Fail. 2019;41(1):507–520. 31216906 10.1080/0886022X.2019.1625787 PMC6586115

[5] Dogan E, Erkoc R, Demir C, et al. Effect of hormone replacement therapy on CD4+ and CD8+ numbers, CD4+/CD8+ ratio, and immunoglobulin levels in hemodialysis patients. LRNF. 2005;27(4):421–424. 16060130

[6] Jacenik D, Beswick EJ, Krajewska WM, et al. G protein-coupled estrogen receptor in colon function, immune regulation and carcinogenesis. World J Gastroenterol. 2019;25(30):4092–4104. 31435166 10.3748/wjg.v25.i30.4092 PMC6700692

[7] Hsu LH, Chu NM, Kao SH. Estrogen, estrogen receptor and lung cancer. Int J Mol Sci. 2017;18(8):1713. 10.3390/ijms18081713 PMC5578103 28783064

[8] Shang DP, Lian HY, Fu DP, et al. Relationship between estrogen receptor 1 gene polymorphisms and postmenopausal osteoporosis of the spine in Chinese women. Genet Mol Res. 2016;15(2):gmr8106. 10.4238/gmr.15028106 27323138

[9] Yang J, Han R, Chen M, et al. Associations of estrogen receptor alpha gene polymorphisms with Type 2 diabetes mellitus and metabolic syndrome: a systematic review and meta-analysis. Horm Metab Res. 2018;50(06):469–477. 29883973 10.1055/a-0620-8553

[10] Tang Y, Min Z, Xiang X-J, et al. Estrogen-related receptor alpha is involved in Alzheimer's disease-like pathology. Exp Neurol. 2018;305:89–96. 29641978 10.1016/j.expneurol.2018.04.003

[11] Muka T, Vargas KG, Jaspers L, et al. Estrogen receptor β actions in the female cardiovascular system: a systematic review of animal and human studies. Maturitas. 2016;86:28–43. 26921926 10.1016/j.maturitas.2016.01.009

[12] Corradetti C, Jog NR, Cesaroni M, et al. Estrogen receptor α signaling exacerbates immune-mediated nephropathies through alteration of metabolic activity. J Immunol. 2018;200(2):512–522. 29237779 10.4049/jimmunol.1700770 PMC5760359

[13] Kline J, Rachoin JS. Acute kidney injury and chronic kidney disease: it's a two-way street. Ren Fail. 2013;35(4):452–455. 23409917 10.3109/0886022X.2013.766572

[14] Chen JQ, Delannoy M, Cooke C, et al. Mitochondrial localization of ERalpha and ERbeta in human MCF7 cells. Am J Physiol Endocrinol Metab. 2004;286(6):E1011–E1022. 14736707 10.1152/ajpendo.00508.2003

[15] Razzaque MA, Masuda N, Maeda Y, et al. Estrogen receptor-related receptor gamma has an exceptionally broad specificity of DNA sequence recognition. Gene. 2004;340(2):275–282. 15475169 10.1016/j.gene.2004.07.010

[16] Guillaume M, Montagner A, Fontaine C, et al. Nuclear and membrane actions of estrogen receptor alpha: contribution to the regulation of energy and glucose homeostasis. Adv Exp Med Biol. 2017;1043:401–426. 29224105 10.1007/978-3-319-70178-3_19

[17] Kim K, Thu N, Saville B, et al. Domains of estrogen receptor alpha (ERalpha) required for ERalpha/Sp1-mediated activation of GC-rich promoters by estrogens and antiestrogens in breast cancer cells. Mol Endocrinol. 2003;17(5):804–817. 12576490 10.1210/me.2002-0406

[18] Sentis S, Le Romancer M, Bianchin C, et al. Sumoylation of the estrogen receptor alpha hinge region regulates its transcriptional activity. Mol Endocrinol. 2005;19(11):2671–2684. 15961505 10.1210/me.2005-0042

[19] Brzozowski AM, Pike AC, Dauter Z, et al. Molecular basis of agonism and antagonism in the oestrogen receptor. Nature. 1997;389(6652):753–758. 9338790 10.1038/39645

[20] Moras D, Gronemeyer H. The nuclear receptor ligand-binding domain: structure and function. Curr Opin Cell Biol. 1998;10(3):384–391. 9640540 10.1016/s0955-0674(98)80015-x

[21] Mosselman S, Polman J, Dijkema R. ER beta: identification and characterization of a novel human estrogen receptor. FEBS Lett. 1996;392(1):49–53. 8769313 10.1016/0014-5793(96)00782-x

[22] Fuentes N, Silveyra P. Estrogen receptor signaling mechanisms. Adv Protein Chem Struct Biol. 2019;116:135–170. 31036290 10.1016/bs.apcsb.2019.01.001 PMC6533072

[23] Hamilton KJ, Hewitt SC, Arao Y, et al. Estrogen hormone biology. Curr Top Dev Biol. 2017;125:109–146. 28527569 10.1016/bs.ctdb.2016.12.005 PMC6206851

[24] Arao Y, Coons LA, Zuercher WJ, et al. Transactivation function-2 of estrogen receptor α contains transactivation function-1-regulating element. J Biol Chem. 2015;290(28):17611–17627. 26028650 10.1074/jbc.M115.638650 PMC4498094

[25] Arao Y, Hamilton KJ, Goulding EH, et al. Transactivating function (AF) 2-mediated AF-1 activity of estrogen receptor α is crucial to maintain male reproductive tract function. Proc Natl Acad Sci USA. 2012;109(51):21140–21145. 23213263 10.1073/pnas.1216189110 PMC3529064

[26] Gosden JR, Middleton PG, Rout D. Localization of the human oestrogen receptor gene to chromosome 6q24–q27 by in situ hybridization. Cytogenet Cell Genet. 1986;43(3–4):218–220. 3802924 10.1159/000132325

[27] Paterni I, Granchi C, Katzenellenbogen JA, et al. Estrogen receptors alpha (ERα) and beta (ERβ): subtype-selective ligands and clinical potential. Steroids. 2014;90:13–29. 24971815 10.1016/j.steroids.2014.06.012 PMC4192010

[28] Chen C, Gong X, Yang X, et al. The roles of estrogen and estrogen receptors in gastrointestinal disease. Oncol Lett. 2019;18(6):5673–5680. 31788039 10.3892/ol.2019.10983 PMC6865762

[29] Wang Z, Zhang X, Shen P, et al. Identification, cloning, and expression of human estrogen receptor-alpha36, a novel variant of human estrogen receptor-alpha66. Biochem Biophys Res Commun. 2005;336(4):1023–1027. 16165085 10.1016/j.bbrc.2005.08.226

[30] Chantalat E, Boudou F, Laurell H, et al. The AF-1-deficient estrogen receptor ERα46 isoform is frequently expressed in human breast tumors. Breast Cancer Res. 2016;18(1):123. 27927249 10.1186/s13058-016-0780-7 PMC5142410

[31] Smith EP, Boyd J, Frank GR, et al. Estrogen resistance caused by a mutation in the estrogen-receptor gene in a man. N Engl J Med. 1994;331(16):1056–1061. 8090165 10.1056/NEJM199410203311604

[32] Riant E, Waget A, Cogo H, et al. Estrogens protect against high-fat diet-induced insulin resistance and glucose intolerance in mice. Endocrinology. 2009;150(5):2109–2117. 19164473 10.1210/en.2008-0971

[33] Yu P, Wang Y, Li C, et al. Protective effects of downregulating estrogen receptor alpha expression in cervical cancer. Anticancer Agents Med Chem. 2018;18(14):1975–1982. 30173650 10.2174/1871520618666180830162517

[34] Pelekanou V, Anastasiou E, Bakogeorgou E, et al. Estrogen receptor-alpha isoforms are the main estrogen receptors expressed in non-small cell lung carcinoma. Steroids. 2019;142:65–76. 29454903 10.1016/j.steroids.2018.01.008

[35] Stein RA, Gaillard S, McDonnell DP. Estrogen-related receptor alpha induces the expression of vascular endothelial growth factor in breast cancer cells. J Steroid Biochem Mol Biol. 2009;114(1–2):106–112. 19429439 10.1016/j.jsbmb.2009.02.010 PMC2680788

[36] Mishra S, Tai Q, Gu X, et al. Estrogen and estrogen receptor alpha promotes malignancy and osteoblastic tumorigenesis in prostate cancer. Oncotarget. 2015;6(42):44388–44402. 26575018 10.18632/oncotarget.6317 PMC4792564

[37] Enmark E, Pelto-Huikko M, Grandien K, et al. Human estrogen receptor beta-gene structure, chromosomal localization, and expression pattern. J Clin Endocrinol Metab. 1997;82(12):4258–4265. 9398750 10.1210/jcem.82.12.4470

[38] Liu J, Sareddy GR, Zhou M, et al. Differential effects of estrogen receptor β isoforms on glioblastoma progression. Cancer Res. 2018;78(12):3176–3189. 29661831 10.1158/0008-5472.CAN-17-3470 PMC6004261

[39] Leung Y-K, Mak P, Hassan S, et al. Estrogen receptor (ER)-beta isoforms: a key to understanding ER-beta signaling. Proc Natl Acad Sci USA. 2006;103(35):13162–13167. 16938840 10.1073/pnas.0605676103 PMC1552044

[40] Fitts JM, Klein RM, Powers CA. Tamoxifen regulation of bone growth and endocrine function in the ovariectomized rat: discrimination of responses involving estrogen receptor α/estrogen receptor β, G protein-coupled estrogen receptor, or estrogen-related receptor γ using fulvestrant (ICI 182780). J Pharmacol Exp Ther. 2011;338(1):246–254. 21464335 10.1124/jpet.110.173955

[41] Younes M, Honma N. Estrogen receptor β. Arch Pathol Lab Med. 2011;135(1):63–66. 21204712 10.5858/2010-0448-RAR.1

[42] Ponnusamy S, Tran QT, Harvey I, et al. Pharmacologic activation of estrogen receptor β increases mitochondrial function, energy expenditure, and brown adipose tissue. Faseb J. 2017;31(1):266–281. 27733447 10.1096/fj.201600787RR PMC5161516

[43] Varshney MK, Inzunza J, Lupu D, et al. Role of estrogen receptor beta in neural differentiation of mouse embryonic stem cells. Proc Natl Acad Sci USA. 2017;114(48): E10428–E10437. 29133394 10.1073/pnas.1714094114 PMC5715781

[44] Efstathiadou ZA, Sakka C, Polyzos SA, et al. Associations of estrogen receptor alpha and Beta gene polymorphisms with lipid levels and insulin resistance in men. Metabolism. 2015;64(5):611–617. 25665486 10.1016/j.metabol.2015.01.006

[45] Edvardsson K, Ström A, Jonsson P, et al. Estrogen receptor β induces antiinflammatory and antitumorigenic networks in colon cancer cells. Mol Endocrinol. 2011;25(6):969–979. 21493669 10.1210/me.2010-0452 PMC5417254

[46] Liu J, Viswanadhapalli S, Garcia L, et al. Therapeutic utility of natural estrogen receptor beta agonists on ovarian cancer. Oncotarget. 2017;8(30):50002–50014. 28654894 10.18632/oncotarget.18442 PMC5564823

[47] Yu C-P, Ho J-Y, Huang Y-T, et al. Estrogen inhibits renal cell carcinoma cell progression through estrogen receptor-β activation. PLOS One. 2013;8(2):e56667. 23460808 10.1371/journal.pone.0056667 PMC3584057

[48] Xiao L, Luo Y, Tai R, et al. Estrogen receptor β suppresses inflammation and the progression of prostate cancer. Mol Med Rep. 2019;19(5):3555–3563. 30864712 10.3892/mmr.2019.10014 PMC6472045

[49] Song P, Li Y, Dong Y, et al. Estrogen receptor β inhibits breast cancer cells migration and invasion through CLDN6-mediated autophagy. J Exp Clin Cancer Res. 2019;38(1):354. 31412908 10.1186/s13046-019-1359-9 PMC6694553

[50] Gustafsson JA, Strom A, Warner M. Update on ERbeta. J Steroid Biochem Mol Biol. 2019;191:105312. 30995525 10.1016/j.jsbmb.2019.02.007

[51] Prossnitz ER, Barton M. Estrogen biology: new insights into GPER function and clinical opportunities. Mol Cell Endocrinol. 2014;389(1–2):71–83. 24530924 10.1016/j.mce.2014.02.002 PMC4040308

[52] Sharma G, Mauvais-Jarvis F, Prossnitz ER. Roles of G protein-coupled estrogen receptor GPER in metabolic regulation. J Steroid Biochem Mol Biol. 2018;176:31–37. 28223150 10.1016/j.jsbmb.2017.02.012 PMC5563497

[53] Barton M, Filardo EJ, Lolait SJ, et al. Twenty years of the G protein-coupled estrogen receptor GPER: historical and personal perspectives. J Steroid Biochem Mol Biol. 2018;176:4–15. 28347854 10.1016/j.jsbmb.2017.03.021 PMC5716468

[54] Gaudet HM, Cheng SB, Christensen EM, et al. The G-protein coupled estrogen receptor, GPER: the inside and inside-out story. Mol Cell Endocrinol. 2015;418 Pt 3(Pt 3):207–219. 10.1016/j.mce.2015.07.016 26190834

[55] Olde B, Leeb-Lundberg LM. GPR30/GPER1: searching for a role in estrogen physiology. Trends Endocrinol Metab. 2009;20(8):409–416. 19734054 10.1016/j.tem.2009.04.006

[56] Zimmerman MA, Budish RA, Kashyap S, et al. GPER-novel membrane oestrogen receptor. Clin Sci. 2016;130(12):1005–1016. 10.1042/CS20160114 PMC5125080 27154744

[57] Meyer MR, Prossnitz ER, Barton M. GPER/GPR30 and regulation of vascular tone and blood pressure. Immunol Endocr Metab Agents Med Chem. 2011;11(4):255–261. 24999376 10.2174/1871522211108040255 PMC4079007

[58] Krejčířová R, Maňasová M, Sommerová V, et al. G protein-coupled estrogen receptor (GPER) in adult boar testes, epididymis and spermatozoa during epididymal maturation. Int J Biol Macromol. 2018;116:113–119. 29730010 10.1016/j.ijbiomac.2018.05.015

[59] Sharma G, Hu C, Brigman JL, et al. GPER deficiency in male mice results in insulin resistance, dyslipidemia, and a proinflammatory state. Endocrinology. 2013;154(11):4136–4145. 23970785 10.1210/en.2013-1357 PMC3800768

[60] Torres-López L, Maycotte P, Liñán-Rico A, et al. Tamoxifen induces toxicity, causes autophagy, and partially reverses dexamethasone resistance in Jurkat T cells. J Leukoc Biol. 2019;105(5):983–998. 30645008 10.1002/JLB.2VMA0818-328R

[61] Haas E, Bhattacharya I, Brailoiu E, et al. Regulatory role of G protein-coupled estrogen receptor for vascular function and obesity. Circ Res. 2009;104(3):288–291. 19179659 10.1161/CIRCRESAHA.108.190892 PMC2782532

[62] Kumar R, Balhuizen A, Amisten S, et al. Insulinotropic and antidiabetic effects of 17β-estradiol and the GPR30 agonist G-1 on human pancreatic islets. Endocrinology. 2011;152(7):2568–2579. 21521748 10.1210/en.2010-1361

[63] Balhuizen A, Kumar R, Amisten S, et al. Activation of G protein-coupled receptor 30 modulates hormone secretion and counteracts cytokine-induced apoptosis in pancreatic islets of female mice. Mol Cell Endocrinol. 2010;320(1–2):16–24. 20122988 10.1016/j.mce.2010.01.030

[64] Feldman RD, Limbird LE. GPER (GPR30): a nongenomic receptor (GPCR) for steroid hormones with implications for cardiovascular disease and cancer. Annu Rev Pharmacol Toxicol. 2017;57:567–584. 27814026 10.1146/annurev-pharmtox-010716-104651

[65] Vrtačnik P, Ostanek B, Mencej-Bedrač S, et al. The many faces of estrogen signaling. Biochem Med. 2014;24(3):329–342. 10.11613/BM.2014.035 PMC4210253 25351351

[66] Wu Q, Chambliss K, Umetani M, et al. Non-nuclear estrogen receptor signaling in the endothelium. J Biol Chem. 2011;286(17):14737–14743. 21343284 10.1074/jbc.R110.191791 PMC3083154

[67] Moriarty K, Kim KH, Bender JR. Minireview: estrogen receptor-mediated rapid signaling. Endocrinology. 2006;147(12):5557–5563. 16946015 10.1210/en.2006-0729

[68] Alexander A, Irving AJ, Harvey J. Emerging roles for the novel estrogen-sensing receptor GPER1 in the CNS. Neuropharmacology. 2017;113(Pt B):652–660. 27392633 10.1016/j.neuropharm.2016.07.003

[69] Björnström L, Sjöberg M. Mechanisms of estrogen receptor signaling: convergence of genomic and nongenomic actions on target genes. Mol Endocrinol. 2005;19(4):833–842. 15695368 10.1210/me.2004-0486

[70] Deroo BJ, Korach KS. Estrogen receptors and human disease. J Clin Invest. 2006;116(3):561–570. 16511588 10.1172/JCI27987 PMC2373424

[71] Nilsson S, Koehler KF. Oestrogen receptors and selective oestrogen receptor modulators: molecular and cellular pharmacology. Basic Clin Pharmacol Toxicol. 2005;96(1):15–25. 15667591 10.1111/j.1742-7843.2005.pto960103.x

[72] Jordan VC. Tamoxifen: catalyst for the change to targeted therapy. Eur J Cancer. 2008;44(1):30–38. 18068350 10.1016/j.ejca.2007.11.002 PMC2566958

[73] Moen MD, Keating GM. Raloxifene: a review of its use in the prevention of invasive breast cancer. Drugs. 2008;68(14):2059–2083. 18778124 10.2165/00003495-200868140-00008

[74] Wardell SE, Nelson ER, Chao CA, et al. Bazedoxifene exhibits antiestrogenic activity in animal models of tamoxifen-resistant breast cancer: implications for treatment of advanced disease. Clin Cancer Res. 2013;19(9):2420–2431. 23536434 10.1158/1078-0432.CCR-12-3771 PMC3643989

[75] Negi S, Koreeda D, Kobayashi S, et al. Acute kidney injury: epidemiology, outcomes, complications, and therapeutic strategies. Semin Dial. 2018;31(5):519–527. 29738093 10.1111/sdi.12705

[76] Fujii T, Uchino S, Takinami M, et al. Subacute kidney injury in hospitalized patients. Clin J Am Soc Nephrol. 2014;9(3):457–461. 24311710 10.2215/CJN.04120413 PMC3944755

[77] Kellum JA, Lameire N, KDIGO AKI Guideline Work Group. Diagnosis, evaluation, and management of acute kidney injury: a KDIGO summary (Part 1). Crit Care. 2013;17(1):204. 23394211 10.1186/cc11454 PMC4057151

[78] Koza Y. Acute kidney injury: current concepts and new insights. J Inj Violence Res. 2016;8(1):58–62. 26804946 10.5249/jivr.v8i1.610 PMC4729334

[79] Neugarten J, Golestaneh L. Female sex reduces the risk of hospital-associated acute kidney injury: a meta-analysis. BMC Nephrol. 2018;19(1):314. 30409132 10.1186/s12882-018-1122-z PMC6225636

[80] Neugarten J, Sandilya S, Singh B, et al. Sex and the risk of AKI following cardio-thoracic surgery: a meta-analysis. CJASN. 2016;11(12):2113–2122. 27797892 10.2215/CJN.03340316 PMC5142065

[81] Diptyanusa A, Phumratanaprapin W, Phonrat B, et al. Characteristics and associated factors of acute kidney injury among adult dengue patients: a retrospective single-center study. PLOS One. 2019;14(1):e0210360. 30615667 10.1371/journal.pone.0210360 PMC6322747

[82] O'Brien Z, Cass A, Cole L, et al. Sex and mortality in septic severe acute kidney injury. J Crit Care. 2019;49:70–76. 30388491 10.1016/j.jcrc.2018.10.017

[83] Vallabhajosyula S, Ya'Qoub L, Dunlay SM, et al. Sex disparities in acute kidney injury complicating acute myocardial infarction with cardiogenic shock. ESC Heart Fail. 2019;6(4):874–877. 31271517 10.1002/ehf2.12482 PMC6676281

[84] Cardinale DA, Larsen FJ, Schiffer TA, et al. Superior intrinsic mitochondrial respiration in women than in men. Front Physiol. 2018;9:1133. 30174617 10.3389/fphys.2018.01133 PMC6108574

[85] Borrás C, Sastre J, García-Sala D, et al. Mitochondria from females exhibit higher antioxidant gene expression and lower oxidative damage than males. Free Radic Biol Med. 2003;34(5):546–552. 12614843 10.1016/s0891-5849(02)01356-4

[86] Bhargava P, Schnellmann RG. Mitochondrial energetics in the kidney. Nat Rev Nephrol. 2017;13(10):629–646. 28804120 10.1038/nrneph.2017.107 PMC5965678

[87] Reed DK, Arany I. p66shc and gender-specific dimorphism in acute renal injury. In Vivo. 2014;28(2):205–208. 24632974

[88] Kang KP, Lee JE, Lee AS, et al. Effect of gender differences on the regulation of renal ischemia-reperfusion-induced inflammation in mice. Mol Med Rep. 2014;9(6):2061–2068. 24682292 10.3892/mmr.2014.2089 PMC4055478

[89] Tanaka R, Yazawa M, Morikawa Y, et al. Sex differences in ischaemia/reperfusion-induced acute kidney injury depends on the degradation of noradrenaline by monoamine oxidase. Clin Exp Pharmacol Physiol. 2017;44(3):371–377. 27998005 10.1111/1440-1681.12713

[90] Hodeify R, Megyesi J, Tarcsafalvi A, et al. Gender differences control the susceptibility to ER stress-induced acute kidney injury. Am J Physiol Renal Physiol. 2013;304(7):F875–F882. 23364800 10.1152/ajprenal.00590.2012 PMC3625845

[91] Woodman AG, Mah R, Keddie D, et al. Prenatal iron deficiency causes sex-dependent mitochondrial dysfunction and oxidative stress in fetal rat kidneys and liver. Faseb J. 2018;32(6):3254–3263. 29401611 10.1096/fj.201701080R

[92] Morigi M, Perico L, Benigni A. Sirtuins in renal health and disease. J Am Soc Nephrol. 2018;29(7):1799–1809. 29712732 10.1681/ASN.2017111218 PMC6050939

[93] Xu S, Gao Y, Zhang Q, et al. SIRT1/3 activation by resveratrol attenuates acute kidney injury in a septic rat model. Oxid Med Cell Longev. 2016;2016:1–12. 10.1155/2016/7296092 PMC5149703 28003866

[94] Ouyang J, Zeng Z, Fang H, et al. SIRT3 inactivation promotes acute kidney injury through elevated acetylation of SOD2 and p53. J Surg Res. 2019;233:221–230. 30502252 10.1016/j.jss.2018.07.019

[95] Ugur S, Ulu R, Dogukan A, et al. The renoprotective effect of curcumin in cisplatin-induced nephrotoxicity. Ren Fail. 2015;37(2):332–336. 10.3109/0886022X.2014.986005 25594614

[96] Wei S, Gao Y, Dai X, et al. SIRT1-mediated HMGB1 deacetylation suppresses sepsis-associated acute kidney injury. Am J Physiol Renal Physiol. 2019;316(1): F20–F31. 30379096 10.1152/ajprenal.00119.2018

[97] Gao Q, Zhu H. The overexpression of Sirtuin1 (SIRT1) alleviated lipopolysaccharide (LPS)-induced Acute Kidney Injury (AKI) via inhibiting the activation of nucleotide-binding oligomerization domain-like receptors (NLR) family pyrin domain containing 3 (NLRP3) inflammasome. Med Sci Monit. 2019;25:2718–2726. 30980521 10.12659/MSM.913146 PMC6476233

[98] Khan M, Ullah R, Rehman SU, et al. 17β-estradiol modulates SIRT1 and halts oxidative stress-mediated cognitive impairment in a male aging mouse model. Cells. 2019;8(8):928. 10.3390/cells8080928 PMC6721687 31430865

[99] Guo J-M, Shu H, Wang L, et al. SIRT1-dependent AMPK pathway in the protection of estrogen against ischemic brain injury. CNS Neurosci Ther. 2017;23(4):360–369. 28256111 10.1111/cns.12686 PMC6492707

[100] Yao Y, Li H, Gu Y, et al. Inhibition of SIRT1 deacetylase suppresses estrogen receptor signaling. Carcinogenesis. 2010;31(3):382–387. 19995796 10.1093/carcin/bgp308 PMC2832546

[101] Malek M, Nematbakhsh M. Renal ischemia/reperfusion injury; from pathophysiology to treatment. J Renal Inj Prev. 2015;4(2):20–27. 26060833 10.12861/jrip.2015.06 PMC4459724

[102] Fu Y, Tang C, Cai J, et al. Rodent models of AKI-CKD transition. Am J Physiol Renal Physiol. 2018;315(4):F1098–F1106. 29949392 10.1152/ajprenal.00199.2018 PMC6230729

[103] Packialakshmi B, Stewart IJ, Burmeister DM, et al. Large animal models for translational research in acute kidney injury. Ren Fail. 2020;42(1):1042–1058. 33043785 10.1080/0886022X.2020.1830108 PMC7586719

[104] Pegues MA, McCrory MA, Zarjou A, et al. C-reactive protein exacerbates renal ischemia-reperfusion injury. Am J Physiol Renal Physiol. 2013;304(11): F1358–F1365. 23535585 10.1152/ajprenal.00476.2012 PMC3680688

[105] Philipponnet C, Aniort J, Garrouste C, et al. Ischemia reperfusion injury in kidney transplantation: a case report. Medicine. 2018;97(52):e13650. 30593134 10.1097/MD.0000000000013650 PMC6314654

[106] Bellomo R, Kellum JA, Ronco C, et al. Acute kidney injury in sepsis. Intensive Care Med. 2017;43(6):816–828. 28364303 10.1007/s00134-017-4755-7

[107] Sandroni C, Dell'anna AM, Tujjar O, et al. Acute kidney injury after cardiac arrest: a systematic review and meta-analysis of clinical studies. Minerva Anestesiol. 2016;82(9):989–999. 26957119

[108] Fu Z-Y, Wu Z-J, Zheng J-H, et al. The incidence of acute kidney injury following cardiac arrest and cardiopulmonary resuscitation in a rat model. Ren Fail. 2019;41(1):278–283. 31014141 10.1080/0886022X.2019.1596819 PMC6493295

[109] Redfield RR, Scalea JR, Zens TJ, et al. Predictors and outcomes of delayed graft function after living-donor kidney transplantation. Transpl Int. 2016;29(1):81–87. 26432507 10.1111/tri.12696

[110] Damodaran S, Bullock B, Ekwenna O, et al. Risk factors for delayed graft function and their impact on graft outcomes in live donor kidney transplantation. Int Urol Nephrol. 2021;53(3):439–446. 33394282 10.1007/s11255-020-02687-5

[111] Lepeytre F, Dahhou M, Zhang X, et al. Association of sex with risk of kidney graft failure differs by age. J Am Soc Nephrol. 2017;28(10):3014–3023. 28592422 10.1681/ASN.2016121380 PMC5619967

[112] Zeier M, Döhler B, Opelz G, et al. The effect of donor gender on graft survival. J Am Soc Nephrol. 2002;13(10):2570–2576. 12239247 10.1097/01.asn.0000030078.74889.69

[113] Aufhauser DD, Wang Z, Murken DR Jr, et al. Improved renal ischemia tolerance in females influences kidney transplantation outcomes. J Clin Invest. 2016;126(5):1968–1977. 27088798 10.1172/JCI84712 PMC4855926

[114] Tanaka R, Tsutsui H, Ohkita M, et al. Sex differences in ischemia/reperfusion-induced acute kidney injury are dependent on the renal sympathetic nervous system. Eur J Pharmacol. 2013;714(1–3):397–404. 23872383 10.1016/j.ejphar.2013.07.008

[115] Tanaka R, Tsutsui H, Kobuchi S, et al. Protective effect of 17β-estradiol on ischemic acute kidney injury through the renal sympathetic nervous system. Eur J Pharmacol. 2012;683(1–3):270–275. 22426161 10.1016/j.ejphar.2012.02.044

[116] Ikeda M, Swide T, Vayl A, et al. Estrogen administered after cardiac arrest and cardiopulmonary resuscitation ameliorates acute kidney injury in a sex- and age-specific manner. Crit Care. 2015;19(1):332. 26384003 10.1186/s13054-015-1049-8 PMC4574460

[117] Hutchens MP, Nakano T, Kosaka Y, et al. Estrogen is renoprotective via a nonreceptor-dependent mechanism after cardiac arrest in vivo. Anesthesiology. 2010;112(2):395–405. 20068453 10.1097/ALN.0b013e3181c98da9 PMC2821813

[118] Park KM, Kim JI, Ahn Y, et al. Testosterone is responsible for enhanced susceptibility of males to ischemic renal injury. J Biol Chem. 2004;279(50):52282–52292. 15358759 10.1074/jbc.M407629200

[119] Singh AP, Singh N, Pathak D, et al. Estradiol attenuates ischemia reperfusion-induced acute kidney injury through PPAR-γ stimulated eNOS activation in rats. Mol Cell Biochem. 2019;453(1–2):1–9. 30194582 10.1007/s11010-018-3427-4

[120] Singh AP, Singh N, Singh Bedi PM. Estrogen attenuates renal IRI through PPAR-γ agonism in rats. J Surg Res. 2016;203(2):324–330. 27363640 10.1016/j.jss.2016.02.038

[121] Liu B, Tan P. PPAR γ/TLR4/TGF-β1 axis mediates the protection effect of erythropoietin on cyclosporin A-induced chronic nephropathy in rat. Ren Fail. 2020;42(1):216–224. 32090669 10.1080/0886022X.2020.1729188 PMC7054967

[122] Žeravica R, Čabarkapa V, Ilinčić B, et al. Plasma endothelin-1 level, measured glomerular filtration rate and effective renal plasma flow in diabetic nephropathy. Ren Fail. 2015;37(4):681–686. 25687384 10.3109/0886022X.2015.1010990

[123] Zager RA, Johnson ACM, Andress D, et al. Progressive endothelin-1 gene activation initiates chronic/end-stage renal disease following experimental ischemic/reperfusion injury. Kidney Int. 2013;84(4):703–712. 23698233 10.1038/ki.2013.157 PMC3788861

[124] Arfian N, Emoto N, Vignon-Zellweger N, et al. ET-1 deletion from endothelial cells protects the kidney during the extension phase of ischemia/reperfusion injury. Biochem Biophys Res Commun. 2012;425(2):443–449. 22846580 10.1016/j.bbrc.2012.07.121

[125] Takaoka M, Yuba M, Fujii T, et al. Oestrogen protects against ischaemic acute renal failure in rats by suppressing renal endothelin-1 overproduction. Clin Sci (Lond). 2002;103 Suppl 48(Suppl 48):434S–437S. 12193139 10.1042/CS103S434S

[126] Ba ZF, Chaudry IH. Role of estrogen receptor subtypes in estrogen-induced organ-specific vasorelaxation after trauma-hemorrhage. Am J Physiol Heart Circ Physiol. 2008;295(5):H2061–H2067. 18805896 10.1152/ajpheart.00707.2008 PMC2614579

[127] Gohar EY, Daugherty EM, Aceves JO, et al. Evidence for G-protein-coupled estrogen receptor as a pronatriuretic factor. J Am Heart Assoc. 2020;9(10):e015110. 32390531 10.1161/JAHA.119.015110 PMC7660860

[128] Wu C-C, Chang C-Y, Chang S-T, et al. 17β-Estradiol accelerated renal tubule regeneration in male rats after ischemia/reperfusion-induced acute kidney injury. Shock. 2016;46(2):158–163. 26849629 10.1097/SHK.0000000000000586

[129] Satake A, Takaoka M, Nishikawa M, et al. Protective effect of 17beta-estradiol on ischemic acute renal failure through the PI3K/Akt/eNOS pathway. Kidney Int. 2008;73(3):308–317. 18004295 10.1038/sj.ki.5002690

[130] Singh AP, Singh N, Bedi PMS. Estradiol mitigates ischemia reperfusion-induced acute renal failure through NMDA receptor antagonism in rats. Mol Cell Biochem. 2017;434(1–2):33–40. 28432550 10.1007/s11010-017-3034-9

[131] Hutchens MP, Fujiyoshi T, Komers R, et al. Estrogen protects renal endothelial barrier function from ischemia-reperfusion in vitro and in vivo. Am J Physiol Renal Physiol. 2012;303(3):F377–F385. 22622457 10.1152/ajprenal.00354.2011 PMC3774263

[132] Chang Y, Han Z, Zhang Y, et al. G protein-coupled estrogen receptor activation improves contractile and diastolic functions in rat renal interlobular artery to protect against renal ischemia reperfusion injury. Biomed Pharmacother = Biomed Pharmacother. 2019;112:108666. 30784936 10.1016/j.biopha.2019.108666

[133] Jørgensen PL. Structure, function and regulation of Na,K-ATPase in the kidney. Kidney Int. 1986;29(1):10–20. 2421041 10.1038/ki.1986.3

[134] Zhang L-M, Jiang L-J, Zhao Z-G, et al. Mesenteric lymph duct ligation after hemorrhagic shock enhances the ATP level and ATPase activity in rat kidneys. Ren Fail. 2014;36(4):593–597. 24742208 10.3109/0886022X.2014.882183

[135] Molinas SM, Trumper L, Serra E, et al. Evolution of renal function and Na+, K +-ATPase expression during ischaemia-reperfusion injury in rat kidney. Mol Cell Biochem. 2006;287(1–2):33–42. 16708288 10.1007/s11010-005-9021-6

[136] Kumaş M, Eşrefoğlu M, Karataş E, et al. Investigation of dose-dependent effects of berberine against renal ischemia/reperfusion injury in experimental diabetic rats. Nefrologia. 2019;39(4):411–423. 30712966 10.1016/j.nefro.2018.10.006

[137] Fekete A, Vannay A, Vér A, et al. Sex differences in the alterations of Na(+), K(+)-ATPase following ischaemia-reperfusion injury in the rat kidney. J Physiol. 2004;555(Pt 2):471–480. 14673189 10.1113/jphysiol.2003.054825 PMC1664838

[138] Gracelli JB, Souza-Menezes J, Barbosa CML, et al. Role of estrogen and progesterone in the modulation of CNG-A1 and Na/K+-ATPase expression in the renal cortex. Cell Physiol Biochem. 2012;30(1):160–172. 22759964 10.1159/000339055

[139] Buléon M, Cuny M, Grellier J, et al. A single dose of estrogen during hemorrhagic shock protects against kidney injury whereas estrogen restoration in ovariectomized mice is ineffective. Sci Rep. 2020;10(1):17240. 33057080 10.1038/s41598-020-73974-5 PMC7560623

[140] Sun J, Zhang J, Tian J, et al. Mitochondria in sepsis-induced AKI. J Am Soc Nephrol. 2019;30(7):1151–1161. 31076465 10.1681/ASN.2018111126 PMC6622414

[141] Vincent J-L, Sakr Y, Sprung CL, et al. Sepsis in European intensive care units: results of the SOAP study. Crit Care Med. 2006;34(2):344–353. 16424713 10.1097/01.ccm.0000194725.48928.3a

[142] Xu X, Nie S, Liu Z, et al. Epidemiology and clinical correlates of AKI in Chinese hospitalized adults. Clin J Am Soc Nephrol. 2015;10(9):1510–1518. 26231194 10.2215/CJN.02140215 PMC4559507

[143] Feng J-Y, Liu K-T, Abraham E, et al. Serum estradiol levels predict survival and acute kidney injury in patients with septic shock–a prospective study. PLoS One. 2014;9(6):e97967. 24904990 10.1371/journal.pone.0097967 PMC4048195

[144] Trentzsch H, Nienaber U, Behnke M, et al. Female sex protects from organ failure and sepsis after major trauma haemorrhage. Injury. 2014;45(Suppl 3):S20–S28. 25284229 10.1016/j.injury.2014.08.013

[145] Chung M-T, Lee Y-M, Shen H-H, et al. Activation of autophagy is involved in the protective effect of 17β-oestradiol on endotoxaemia-induced multiple organ dysfunction in ovariectomized rats. J Cell Mol Med. 2017;21(12):3705–3717. 28714586 10.1111/jcmm.13280 PMC5706505

[146] Shen H-H, Huang S-Y, Cheng P-Y, et al. Involvement of HSP70 and HO-1 in the protective effects of raloxifene on multiple organ dysfunction syndrome by endotoxemia in ovariectomized rats. Menopause. 2017;24(8):959–969. 28350760 10.1097/GME.0000000000000864

[147] Zhong L, Zhou X-L, Liu Y-S, et al. Estrogen receptor α mediates the effects of notoginsenoside R1 on endotoxin-induced inflammatory and apoptotic responses in H9c2 cardiomyocytes. Mol Med Rep. 2015;12(1):119–126. 25738436 10.3892/mmr.2015.3394 PMC4438911

[148] Yuk J-M, Kim TS, Kim SY, et al. Orphan nuclear receptor ERRα controls macrophage metabolic signaling and A20 expression to negatively regulate TLR-induced inflammation. Immunity. 2015;43(1):80–91. 26200012 10.1016/j.immuni.2015.07.003

[149] Christaki E, Opal SM, Keith JC, et al. Estrogen receptor beta agonism increases survival in experimentally induced sepsis and ameliorates the genomic sepsis signature: a pharmacogenomic study. J Infect Dis. 2010;201(8):1250–1257. 20205571 10.1086/651276

[150] Bösch F, Angele MK, Chaudry IH. Gender differences in trauma, shock and sepsis. Mil Med Res. 2018;5(1):35. 30360757 10.1186/s40779-018-0182-5 PMC6203206

[151] Kawasaki T, Chaudry IH. The effects of estrogen on various organs: therapeutic approach for sepsis, trauma, and reperfusion injury. Part 2: liver, intestine, spleen, and kidney. J Anesth. 2012;26(6):892–899. 22729228 10.1007/s00540-012-1426-2

[152] Srisawat N, Kulvichit W, Mahamitra N, et al. The epidemiology and characteristics of acute kidney injury in the Southeast Asia intensive care unit: a prospective multicentre study. Nephrol Dial Transplant. 2019; 35(10):1729–1738. 10.1093/ndt/gfz087 31075172

[153] Goswami S, Pahwa N, Vohra R, et al. Clinical spectrum of hospital acquired acute kidney injury: a prospective study from Central India. Saudi J Kidney Dis Transpl. 2018;29(4):946–955. 30152434 10.4103/1319-2442.239650

[154] Mehta RL, Pascual MT, Soroko S, et al. Spectrum of acute renal failure in the intensive care unit: the PICARD experience. Kidney Int. 2004;66(4):1613–1621. 15458458 10.1111/j.1523-1755.2004.00927.x

[155] Perazella MA. Pharmacology behind common drug nephrotoxicities. Clin J Am Soc Nephrol. 2018;13(12):1897–1908. 29622670 10.2215/CJN.00150118 PMC6302342

[156] Perazella MA. Drug-induced acute kidney injury: diverse mechanisms of tubular injury. Curr Opin Crit Care. 2019;25(6):550–557. 31483318 10.1097/MCC.0000000000000653

[157] Hosohata K. Role of oxidative stress in drug-induced kidney injury. Int J Mol Sci. 2016;17(11): 1826. 10.3390/ijms17111826 PMC5133827 27809280

[158] Muriithi AK, Leung N, Valeri AM, et al. Biopsy-proven acute interstitial nephritis, 1993–2011: a case series. Am J Kidney Dis. 2014;64(4):558–566. 24927897 10.1053/j.ajkd.2014.04.027

[159] Muriithi AK, Leung N, Valeri AM, et al. Clinical characteristics, causes and outcomes of acute interstitial nephritis in the elderly. Kidney Int. 2015;87(2):458–464. 25185078 10.1038/ki.2014.294

[160] Sweileh WM. Gender differences in aminoglycoside induced nephrotoxicity: a prospective, hospital-based study. Curr Clin Pharmacol. 2009;4(3):229–232. 19500072 10.2174/157488409789375339

[161] Chen W-Y, Hsiao C-H, Chen Y-C, et al. Cisplatin nephrotoxicity might have a sex difference. An analysis based on women's sex hormone changes. J Cancer. 2017;8(19):3939–3944. 29187868 10.7150/jca.20083 PMC5705995

[162] Neugarten J, Golestaneh L. The effect of gender on aminoglycoside-associated nephrotoxicity. Clin Nephrol. 2016;86(10):183–189. 27616758 10.5414/CN108927

[163] Zajjari Y, Montasser D, Sobhi A, et al. Acute interstitial nephritis in the military hospital of Morocco: clinical features and renal outcomes. Saudi J Kidney Dis Transpl. 2019;30(6):1407–1414. 31929288 10.4103/1319-2442.275485

[164] Joseph S, Nicolson TJ, Hammons G, et al. Expression of drug transporters in human kidney: impact of sex, age, and ethnicity. Biol Sex Differ. 2015;6(1):4. 25750709 10.1186/s13293-015-0020-3 PMC4352278

[165] Oswald S, Müller J, Neugebauer U, et al. Protein abundance of clinically relevant drug transporters in the human kidneys. Int J Mol Sci. 2019;20(21):5303. 10.3390/ijms20215303 PMC6862022 31653114

[166] Li CY, Hosey-Cojocari C, Basit A, et al. Optimized renal transporter quantification by using Aquaporin 1 and Aquaporin 2 as anatomical markers: application in characterizing the ontogeny of renal transporters and its correlation with hepatic transporters in paired human samples. Aaps J. 2019;21(5):88. 31297641 10.1208/s12248-019-0359-1 PMC7413827

[167] Kwekel JC, Desai VG, Moland CL, et al. Sex differences in kidney gene expression during the life cycle of F344 rats. Biol Sex Differ. 2013;4(1):14. 23902594 10.1186/2042-6410-4-14 PMC3844475

[168] Hazelhoff MH, Bulacio RP, Chevalier A, et al. Renal expression of organic anion transporters is modified after mercuric chloride exposure: gender-related differences. Toxicol Lett. 2018;295:390–396. 30031051 10.1016/j.toxlet.2018.07.016

[169] Ljubojević M, Balen D, Breljak D, et al. Renal expression of organic anion transporter OAT2 in rats and mice is regulated by sex hormones. Am J Physiol Renal Physiol. 2007;292(1): F361–F372. 16885152 10.1152/ajprenal.00207.2006

[170] Nematbakhsh M, Ebrahimian S, Tooyserkani M, et al. Gender difference in Cisplatin-induced nephrotoxicity in a rat model: greater intensity of damage in male than female. Nephro Urol Mon. 2013;5(3):818–821. 10.5812/numonthly.10128 PMC3830908 24282792

[171] Boddu R, Fan C, Rangarajan S, et al. Unique sex- and age-dependent effects in protective pathways in acute kidney injury. Am J Physiol Renal Physiol. 2017;313(3): F740–F755. 28679590 10.1152/ajprenal.00049.2017 PMC5625098

[172] Kurt AH, Bozkus F, Uremis N, et al. The protective role of G protein-coupled estrogen receptor 1 (GPER-1) on methotrexate-induced nephrotoxicity in human renal epithelium cells. Ren Fail. 2016;38(5):686–692. 26981789 10.3109/0886022X.2016.1155398

[173] Hernández-Esquivel L, Zazueta C, Buelna-Chontal M, et al. Protective behavior of tamoxifen against Hg2+-induced toxicity on kidney mitochondria: in vitro and in vivo experiments. J Steroid Biochem Mol Biol. 2011;127(3–5):345–350. 21821123 10.1016/j.jsbmb.2011.07.004

[174] Abd El-Lateef SM, El-Sayed E-SM, Mansour AM, et al. The protective role of estrogen and its receptors in gentamicin-induced acute kidney injury in rats. Life Sci. 2019;239: 117082. 31756345 10.1016/j.lfs.2019.117082

[175] Lee CI, Goodwin A, Wilcken N. Fulvestrant for hormone-sensitive metastatic breast cancer. Cochrane Database Syst Rev. 2017;1(1):CD011093. 28043088 10.1002/14651858.CD011093.pub2 PMC6464820

[176] Ghane Shahrbaf F, Assadi F. Drug-induced renal disorders. J Renal Inj Prev. 2015;4(3):57–60. 26468475 10.12861/jrip.2015.12 PMC4594214

[177] Katarey D, Verma S. Drug-induced liver injury. Clin Med (Lond). 2016;16(Suppl 6):s104–s109. 27956449 10.7861/clinmedicine.16-6s-s104 PMC6329561

[178] Danese E, Montagnana M, Favaloro EJ, et al. Drug-induced thrombocytopenia: mechanisms and laboratory diagnostics. Semin Thromb Hemost. 2020;46(03):264–274. 31563127 10.1055/s-0039-1697930

[179] Kidney Disease: Improving Global Outcomes (KDIGO) CKD Work Group. KDIGO 2012 clinical practice guideline for the evaluation and management of chronic kidney disease. Kidney Int Suppl. 2013;3(1):1–150.

[180] Chen L, Luo M, Dong C, et al. Pathological spectrum of glomerular disease in patients with renal insufficiency: a single-center study in Northeastern China. Ren Fail. 2019;41(1):473–480. 31198075 10.1080/0886022X.2019.1620774 PMC6586151

[181] Xie Y, Chen X. Epidemiology, major outcomes, risk factors, prevention and management of chronic kidney disease in China. Am J Nephrol. 2008;28(1):1–7. 17890852 10.1159/000108755

[182] Halbesma N, Brantsma AH, Bakker SJL, et al. Gender differences in predictors of the decline of renal function in the general population. Kidney Int. 2008;74(4):505–512. 18496511 10.1038/ki.2008.200

[183] Eriksen BO, Ingebretsen OC. The progression of chronic kidney disease: a 10-year population-based study of the effects of gender and age. Kidney Int. 2006;69(2):375–382. 16408129 10.1038/sj.ki.5000058

[184] Kattah AG, Smith CY, Gazzuola Rocca L, et al. CKD in patients with bilateral oophorectomy. CJASN. 2018;13(11):1649–1658. 30232136 10.2215/CJN.03990318 PMC6237067

[185] Hecking M, Bieber BA, Ethier J, et al. Sex-specific differences in hemodialysis prevalence and practices and the male-to-female mortality rate: the Dialysis Outcomes and Practice Patterns Study (DOPPS). PLoS Med. 2014;11(10):e1001750. 25350533 10.1371/journal.pmed.1001750 PMC4211675

[186] Carrero JJ, Hecking M, Chesnaye NC, et al. Sex and gender disparities in the epidemiology and outcomes of chronic kidney disease. Nat Rev Nephrol. 2018;14(3):151–164. 29355169 10.1038/nrneph.2017.181

[187] Matuszkiewicz-Rowinska J, Skorzewska K, Radowicki S, et al. Endometrial morphology and pituitary-gonadal axis dysfunction in women of reproductive age undergoing chronic haemodialysis–a multicentre study. Nephrol Dial Transplant. 2004;19(8):2074–2077. 15173376 10.1093/ndt/gfh279

[188] Park YJ, Kim JM. Klotho and postmenopausal hormone replacement therapy in women with chronic kidney disease. J Menopausal Med. 2018;24(2):75–80. 30202755 10.6118/jmm.2018.24.2.75 PMC6127018

[189] Ji H, Pesce C, Zheng W, et al. Sex differences in renal injury and nitric oxide production in renal wrap hypertension. Am J Physiol Heart Circ Physiol. 2005;288(1):H43–H47. 15319201 10.1152/ajpheart.00630.2004

[190] Ji H, Zheng W, Menini S, et al. Female protection in progressive renal disease is associated with estradiol attenuation of superoxide production. Gend Med. 2007;4(1):56–71. 17584628 10.1016/s1550-8579(07)80009-x

[191] Flyvbjerg A. The role of the complement system in diabetic nephropathy. Nat Rev Nephrol. 2017;13(5):311–318. 28262777 10.1038/nrneph.2017.31

[192] Möllsten A, Svensson M, Waernbaum I, et al. Cumulative risk, age at onset, and sex-specific differences for developing end-stage renal disease in young patients with type 1 diabetes: a nationwide population-based cohort study. Diabetes. 2010;59(7):1803–1808. 20424230 10.2337/db09-1744 PMC2889782

[193] Goñi MJ, Forga L, Ibañez B, et al. Incidence and risk factors involved in the development of nephropathy in patients with Type 1 diabetes mellitus: follow up since onset. Can J Diabetes. 2016;40(3):258–263. 26976719 10.1016/j.jcjd.2015.11.008

[194] Costacou T, Orchard TJ. Cumulative kidney complication risk by 50 years of Type 1 diabetes: the effects of sex, age, and calendar year at onset. Diabetes Care. 2018;41(3):426–433. 28931542 10.2337/dc17-1118 PMC5829956

[195] Maric-Bilkan C. Sex differences in diabetic kidney disease. Mayo Clin Proc. 2020;95(3):587–599. 32138885 10.1016/j.mayocp.2019.08.026

[196] Maric C, Forsblom C, Thorn L, et al. Association between testosterone, estradiol and sex hormone binding globulin levels in men with type 1 diabetes with nephropathy. Steroids. 2010;75(11):772–778. 20105436 10.1016/j.steroids.2010.01.011 PMC2891875

[197] Salonia A, Lanzi R, Scavini M, et al. Sexual function and endocrine profile in fertile women with type 1 diabetes. Diabetes Care. 2006;29(2):312–316. 16443879 10.2337/diacare.29.02.06.dc05-1067

[198] Inada A, Inada O, Fujii NL, et al. Adjusting the 17β-estradiol-to-androgen ratio ameliorates diabetic nephropathy. J Am Soc Nephrol. 2016;27(10):3035–3050. 26940099 10.1681/ASN.2015070741 PMC5042662

[199] Mankhey RW, Bhatti F, Maric C. 17beta-Estradiol replacement improves renal function and pathology associated with diabetic nephropathy. Am J Physiol Renal Physiol. 2005;288(2):F399–F405. 15454392 10.1152/ajprenal.00195.2004

[200] Riazi S, Maric C, Ecelbarger CA. 17-beta Estradiol attenuates streptozotocin-induced diabetes and regulates the expression of renal sodium transporters. Kidney Int. 2006;69(3):471–480. 16514430 10.1038/sj.ki.5000140

[201] Dixon A, Wells CC, Singh S, et al. Renoprotective effects of a selective estrogen receptor modulator, raloxifene, in an animal model of diabetic nephropathy. Am J Nephrol. 2007;27(2):120–128. 17308373 10.1159/000099837 PMC3179626

[202] Ulas M, Cay M. 17β-Estradiol and vitamin E modulates oxidative stress-induced kidney toxicity in diabetic ovariectomized rat. Biol Trace Elem Res. 2011;144(1–3):821–831. 21484408 10.1007/s12011-011-9025-x

[203] Tomiyoshi Y, Sakemi T, Aoki S, et al. Different effects of castration and estrogen administration on glomerular injury in spontaneously hyperglycemic Otsuka Long-Evans Tokushima Fatty (OLETF) rats. Nephron. 2002;92(4):860–867. 12399633 10.1159/000065442

[204] Mankhey RW, Wells CC, Bhatti F, et al. 17beta-Estradiol supplementation reduces tubulointerstitial fibrosis by increasing MMP activity in the diabetic kidney. Am J Physiol Regul Integr Comp Physiol. 2007;292(2):R769–R77. 16931652 10.1152/ajpregu.00375.2006

[205] Dixon A, Maric C. 17beta-Estradiol attenuates diabetic kidney disease by regulating extracellular matrix and transforming growth factor-beta protein expression and signaling. Am J Physiol Renal Physiol. 2007;293(5):F1678–F1690. 17686959 10.1152/ajprenal.00079.2007 PMC3179625

[206] Chin M, Isono M, Isshiki K, et al. Estrogen and raloxifene, a selective estrogen receptor modulator, ameliorate renal damage in db/db mice. Am J Pathol. 2005;166(6):1629–1636. 15920148 10.1016/s0002-9440(10)62473-x PMC1602422

[207] Keene KL, Mychaleckyj JC, Smith SG, et al. Comprehensive evaluation of the estrogen receptor alpha gene reveals further evidence for association with type 2 diabetes enriched for nephropathy in an African American population. Hum Genet. 2008;123(4):333–341. 18305958 10.1007/s00439-008-0482-z PMC2752813

[208] Ryba-Stanisławowska M, Rybarczyk-Kapturska K, Brandt A, et al. IVS1 -397T > C estrogen receptor α polymorphism is associated with low-grade systemic inflammatory response in type 1 diabetic girls. Mediators Inflamm. 2014;2014:839585. 24523574 10.1155/2014/839585 PMC3910071

[209] Irsik DL, Romero-Aleshire MJ, Chavez EM, et al. Renoprotective impact of estrogen receptor-α and its splice variants in female mice with type 1 diabetes. Am J Physiol Renal Physiol. 2018;315(3):F512–F520. 29667912 10.1152/ajprenal.00231.2017 PMC6842867

[210] Wang XX, Wang D, Luo Y, et al. FXR/TGR5 dual agonist prevents progression of nephropathy in diabetes and obesity. J Am Soc Nephrol. 2018;29(1):118–137. 29089371 10.1681/ASN.2017020222 PMC5748904

[211] Tung C-W, Hsu Y-C, Shih Y-H, et al. Glomerular mesangial cell and podocyte injuries in diabetic nephropathy. Nephrology (Carlton). 2018;23 (Suppl 4):32–37. 30298646 10.1111/nep.13451

[212] Catanuto P, Xia X, Pereira-Simon S, et al. Estrogen receptor subtype ratio change protects against podocyte damage. Curr Trends Endocinol. 2017;9:19–29. 29367812 PMC5777622

[213] Potier M, Elliot SJ, Tack I, et al. Expression and regulation of estrogen receptors in mesangial cells: influence on matrix metalloproteinase-9. J Am Soc Nephrol. 2001;12(2):241–251. 11158214 10.1681/ASN.V122241

[214] Ren W, Yi H, Bao Y, et al. Oestrogen inhibits PTPRO to prevent the apoptosis of renal podocytes. Exp Ther Med. 2019;17(3):2373–2380. 30783489 10.3892/etm.2019.7167 PMC6364249

[215] Catanuto P, Doublier S, Lupia E, et al. 17 beta-estradiol and tamoxifen upregulate estrogen receptor beta expression and control podocyte signaling pathways in a model of type 2 diabetes. Kidney Int. 2009;75(11):1194–1201. 19279558 10.1038/ki.2009.69

[216] Catanuto P, Fornoni A, Pereira-Simon S, et al. In vivo 17β-estradiol treatment contributes to podocyte actin stabilization in female db/db mice. Endocrinology. 2012;153(12):5888–5895. 23070549 10.1210/en.2012-1637 PMC3512061

[217] Kummer S, Jeruschke S, Wegerich LV, et al. Estrogen receptor alpha expression in podocytes mediates protection against apoptosis in-vitro and in-vivo. PLoS One. 2011;6(11):e27457. 22096576 10.1371/journal.pone.0027457 PMC3214053

[218] Doublier S, Lupia E, Catanuto P, et al. Testosterone and 17β-estradiol have opposite effects on podocyte apoptosis that precedes glomerulosclerosis in female estrogen receptor knockout mice. Kidney Int. 2011;79(4):404–413. 20962747 10.1038/ki.2010.398 PMC4775100

[219] Qi MY, Chen K, Liu HR, et al. Protective effect of Icariin on the early stage of experimental diabetic nephropathy induced by streptozotocin via modulating transforming growth factor β1 and type IV collagen expression in rats. J Ethnopharmacol. 2011;138(3):731–736. 22027446 10.1016/j.jep.2011.10.015

[220] Li Y-C, Ding X-S, Li H-M, et al. Role of G protein-coupled estrogen receptor 1 in modulating transforming growth factor-β stimulated mesangial cell extracellular matrix synthesis and migration. Mol Cell Endocrinol. 2014;391(1–2):50–59. 24793639 10.1016/j.mce.2014.04.014

[221] Li Y-C, Ding X-S, Li H-M, et al. Icariin attenuates high glucose-induced type IV collagen and fibronectin accumulation in glomerular mesangial cells by inhibiting transforming growth factor-β production and signalling through G protein-coupled oestrogen receptor 1. Clin Exp Pharmacol Physiol. 2013;40(9):635–643. 23772748 10.1111/1440-1681.12143

[222] Qiao C, Ye W, Li S, et al. Icariin modulates mitochondrial function and apoptosis in high glucose-induced glomerular podocytes through G protein-coupled estrogen receptors. Mol Cell Endocrinol. 2018;473:146–155. 29373840 10.1016/j.mce.2018.01.014

[223] Clotet-Freixas S, Soler MJ, Palau V, et al. Sex dimorphism in ANGII-mediated crosstalk between ACE2 and ACE in diabetic nephropathy. Lab Invest. 2018;98(9):1237–1249. 29884907 10.1038/s41374-018-0084-x

[224] de Alencar Franco Costa D, Todiras M, Campos LA, et al. Sex-dependent differences in renal angiotensinogen as an early marker of diabetic nephropathy. Acta Physiol. 2015;213(3):740–746. 10.1111/apha.12441 25529203

[225] Almaani S, Meara A, Rovin BH. Update on lupus nephritis. Clin J Am Soc Nephrol. 2017;12(5):825–835. 27821390 10.2215/CJN.05780616 PMC5477208

[226] Margery-Muir AA, Bundell C, Nelson D, et al. Gender balance in patients with systemic lupus erythematosus. Autoimmun Rev. 2017;16(3):258–268. 28137478 10.1016/j.autrev.2017.01.007

[227] Guéry JC. Why is systemic lupus erythematosus more common in women? Joint Bone Spine. 2019;86(3):297–299. 30584922 10.1016/j.jbspin.2018.12.004

[228] Rojas-Villarraga AJV, Torres-Gonzalez M, Ruiz-Sternberg Á. Safety of hormonal replacement therapy and oral contraceptives in systemic lupus erythematosus: a systematic review and meta-analysis. PLoS One. 2014;9(8):e104303. 25137236 10.1371/journal.pone.0104303 PMC4138076

[229] Kovats S. Estrogen receptors regulate innate immune cells and signaling pathways. Cell Immunol. 2015;294(2):63–69. 25682174 10.1016/j.cellimm.2015.01.018 PMC4380804

[230] Moulton VR. Sex hormones in acquired immunity and autoimmune disease. Front Immunol. 2018;9:2279. 30337927 10.3389/fimmu.2018.02279 PMC6180207

[231] Kim W-U, Min S-Y, Hwang S-H, et al. Effect of oestrogen on T cell apoptosis in patients with systemic lupus erythematosus. Clin Exp Immunol. 2010;161(3):453–458. 20529085 10.1111/j.1365-2249.2010.04194.x PMC2962962

[232] Rider V, Jones S, Evans M, et al. Estrogen increases CD40 ligand expression in T cells from women with systemic lupus erythematosus. J Rheumatol. 2001;28(12):2644–2649. 11764210

[233] Grimaldi CM, Jeganathan V, Diamond B. Hormonal regulation of B cell development: 17 beta-estradiol impairs negative selection of high-affinity DNA-reactive B cells at more than one developmental checkpoint. J Immunol. 2006;176(5):2703–2710. 16493025 10.4049/jimmunol.176.5.2703

[234] Grimaldi CM. Sex and systemic lupus erythematosus: the role of the sex hormones estrogen and prolactin on the regulation of autoreactive B cells. Curr Opin Rheumatol. 2006;18(5):456–461. 16896282 10.1097/01.bor.0000240354.37927.dd

[235] Fan H, Dong G, Zhao G, et al. Gender differences of B cell signature in healthy subjects underlie disparities in incidence and course of SLE related to estrogen. J Immunol Res. 2014;2014:814598. 24741625 10.1155/2014/814598 PMC3987971

[236] Fan H, Zhao G, Ren D, et al. Gender differences of B cell signature related to estrogen-induced IFI44L/BAFF in systemic lupus erythematosus. Immunol Lett. 2017;181:71–78. 27923569 10.1016/j.imlet.2016.12.002

[237] Ramanujam M, Steffgen J, Visvanathan S, et al. Phoenix from the flames: rediscovering the role of the CD40-CD40L pathway in systemic lupus erythematosus and lupus nephritis. Autoimmun Rev. 2020;19(11):102668. 32942031 10.1016/j.autrev.2020.102668

[238] Abdou NI, Rider V, Greenwell C, et al. Fulvestrant (Faslodex), an estrogen selective receptor downregulator, in therapy of women with systemic lupus erythematosus. clinical, serologic, bone density, and T cell activation marker studies: a double-blind placebo-controlled trial. J Rheumatol. 2008;35(5):797. 18381791

[239] Ding Y, He J, Guo J-P, et al. Gender differences are associated with the clinical features of systemic lupus erythematosus. Chin Med J (Engl). 2012;125(14):2477–2481. 22882925

[240] Faezi ST, Hosseini Almodarresi M, Akbarian M, et al. Clinical and immunological pattern of systemic lupus erythematosus in men in a cohort of 2355 patients. Int J Rheum Dis. 2014;17(4):394–399. 24618453 10.1111/1756-185X.12268

[241] Alonso MD, Martínez-Vázquez F, Riancho-Zarrabeitia L, et al. Sex differences in patients with systemic lupus erythematosus from Northwest Spain. Rheumatol Int. 2014;34(1):11–24. 23812032 10.1007/s00296-013-2798-9

[242] Zhang S, Su J, Li X, CSTAR Co-authors, et al. Chinese SLE Treatment and Research group (CSTAR) registry: V. gender impact on Chinese patients with systemic lupus erythematosus. Lupus. 2015;24(12):1267–1275. 25972364 10.1177/0961203315585813

[243] Rastin M, Mahmoudi M, Sahebari M, et al. Clinical & immunological characteristics in systemic lupus erythematosus patients. Indian J Med Res. 2017;146(2):224–229. 29265023 10.4103/ijmr.IJMR_1356_15 PMC5761032

[244] Urrestarazú A, Otatti G, Silvariño R, et al. Lupus nephritis in males: clinical features, course, and prognostic factors for end-stage renal disease. Kidney Int Rep. 2017;2(5):905–912. 29270496 10.1016/j.ekir.2017.05.011 PMC5733876

[245] Santamaría-Alza Y, Motta JZN, Fajardo-Rivero JE, et al. Systemic lupus erythematosus, gender differences in Colombian patients. Clin Rheumatol. 2018;37(9):2423–2428. 29860566 10.1007/s10067-018-4161-8

[246] De Oliveira NT, Silva NG, Dos Santos TAFG, Department of Medicine, Mackenzie Evangelical School of Medicine, Curitiba, Brazil, et al. Clinical and autoantibody profile in male and female patients with systemic lupus erythematosus: a retrospective study in 603 Brazilian patients. Eur J Rheumatol. 2020;7(4):164–168. 10.5152/eurjrheum.2020.20023 PMC7574763 32910762

[247] Hwang J, Lee J, Ahn JK, et al. Clinical characteristics of male and female Korean patients with systemic lupus erythematosus: a comparative study. Korean J Intern Med. 2015;30(2):242–249. 25750567 10.3904/kjim.2015.30.2.242 PMC4351332

[248] Van Griensven M, Bergijk EC, Baelde JJ, et al. Differential effects of sex hormones on autoantibody production and proteinuria in chronic graft-versus-host disease-induced experimental lupus nephritis. Clin Exp Immunol. 1997;107(2):254–260. 9030861 10.1111/j.1365-2249.1997.261-ce1141.x PMC1904571

[249] Bassi N, Luisetto R, Ghirardello A, et al. 17-β-estradiol affects BLyS serum levels and the nephritogenic autoantibody network accelerating glomerulonephritis in NZB/WF1 mice. Lupus. 2015;24(4–5):382–391. 25801881 10.1177/0961203314559636

[250] Feng F, Nyland J, Banyai M, et al. The induction of the lupus phenotype by estrogen is via an estrogen receptor-alpha-dependent pathway. Clin Immunol. 2010;134(2):226–236. 19926524 10.1016/j.clim.2009.10.004

[251] Feng F, Silvin CJ, Fiore NC, et al. 17β-Estradiol (E-2) administration to male (NZB × SWR)F_1_ mice results in increased Id(LN)F_1_-reactive memory T-lymphocytes and accelerated glomerulonephritis. Lupus. 2012;21(3):288–301. 22065096 10.1177/0961203311425519

[252] Yurino H, Ishikawa S, Sato T, et al. Endocrine disruptors (environmental estrogens) enhance autoantibody production by B1 cells. Toxicol Sci. 2004;81(1):139–147. 15166399 10.1093/toxsci/kfh179

[253] Tabor DE, Gould KA. Estrogen receptor alpha promotes lupus in (NZB × NZW)F1 mice in a B cell intrinsic manner. Clin Immunol. 2017;174:41–52. 27989899 10.1016/j.clim.2016.10.011 PMC5316311

[254] Bynoté KK, Hackenberg JM, Korach KS, et al. Estrogen receptor-alpha deficiency attenuates autoimmune disease in (NZB x NZW)F1 mice. Genes Immun. 2008;9(2):137–152. 18200028 10.1038/sj.gene.6364458

[255] Svenson JL, EuDaly J, Ruiz P, et al. Impact of estrogen receptor deficiency on disease expression in the NZM2410 lupus prone mouse. Clin Immunol. 2008;128(2):259–268. 18514033 10.1016/j.clim.2008.03.508 PMC4778964

[256] Cunningham MA, Richard ML, Wirth JR, et al. Novel mechanism for estrogen receptor alpha modulation of murine lupus. J Autoimmun. 2019;97:59–69. 30416032 10.1016/j.jaut.2018.10.011 PMC6351212

[257] Scott JL, Wirth JR, Eudaly J, et al. Complete knockout of estrogen receptor alpha is not directly protective in murine lupus. Clin Immunol. 2017;183:132–141. 28822833 10.1016/j.clim.2017.08.010 PMC6261466

[258] Shim G-J, Kis LL, Warner M, et al. Autoimmune glomerulonephritis with spontaneous formation of splenic germinal centers in mice lacking the estrogen receptor alpha gene. Proc Natl Acad Sci U S A. 2004;101(6):1720–1724. 14745006 10.1073/pnas.0307915100 PMC341834

[259] Xie Q-M, Hu H-Q, Li S-S, et al. Association of oestrogen receptor alpha gene polymorphisms with systemic lupus erythematosus risk: an updated meta-analysis. Microb Pathog. 2019;127:352–358. 30572014 10.1016/j.micpath.2018.12.029

[260] Drehmer MN, Andrade D, Pereira IA, et al. Estrogen receptor alpha gene ( ESR1) polymorphism can contribute to clinical findings in systemic lupus erythematosus patients. Lupus. 2017;26(3):294–298. 27681518 10.1177/0961203316668041

[261] Liu Z-H, Cheng Z-H, Gong R-J, et al. Sex differences in estrogen receptor gene polymorphism and its association with lupus nephritis in Chinese. Nephron. 2002;90(2):174–180. 11818702 10.1159/000049039

[262] Lee MH, Chakhtoura M, Sriram U, et al. Conventional DCs from male and female lupus-prone B6.NZM Sle1/Sle2/Sle3 mice express an IFN signature and have a higher immunometabolism that are enhanced by estrogen. J Immunol Res. 2018;2018:1601079. 29850618 10.1155/2018/1601079 PMC5925037

[263] Cunningham MA, Naga OS, Eudaly JG, et al. Estrogen receptor alpha modulates Toll-like receptor signaling in murine lupus. Clin Immunol. 2012;144(1):1–12. 22659029 10.1016/j.clim.2012.04.001 PMC3737583

[264] Svenson J, Cunningham M, Dasgupta S, et al. Estrogen receptor alpha modulates mesangial cell responses to toll-like receptor ligands. Am J Med Sci. 2014;348(6):492–500. 25343264 10.1097/MAJ.0000000000000339

[265] Xue L, Liu Z, Hu J, et al. Estrogen-induced expression of tumor necrosis factor-like weak inducer of apoptosis through ERα accelerates the progression of lupus nephritis. Rheumatology (Oxford). 2016;55(10):1880–1888. 27354685 10.1093/rheumatology/kew248

[266] Rider V, Abdou NI, Kimler BF, et al. Gender bias in human systemic lupus erythematosus: a problem of steroid receptor action? Front Immunol. 2018;9:611. 29643853 10.3389/fimmu.2018.00611 PMC5882779

[267] Rodrigues JC, Haas M, Reich HN. IgA nephropathy. Clin J Am Soc Nephrol. 2017;12(4):677–686. 28159829 10.2215/CJN.07420716 PMC5383386

[268] Huang PP, Shu DH, Su Z, et al. Association between lifestyle, gender and risk for developing end-stage renal failure in IgA nephropathy: a case-control study within 10 years. Ren Fail. 2019;41(1):914–920. 31580172 10.1080/0886022X.2019.1635029 PMC6781456

[269] Deng W, Tan X, Zhou Q, et al. Gender-related differences in clinicopathological characteristics and renal outcomes of Chinese patients with IgA nephropathy. BMC Nephrol. 2018;19(1):31. 29415664 10.1186/s12882-018-0829-1 PMC5804055

[270] Greene DM, Azcona-Olivera JI, Pestka JJ. Vomitoxin (deoxynivalenol)-induced IgA nephropathy in the B6C3F1 mouse: dose response and male predilection. Toxicology. 1994;92(1–3):245–260. 7940564 10.1016/0300-483x(94)90181-3

[271] Greene DM, Azcona-Olivera JI, Murtha JM, et al. Effects of dihydrotestosterone and estradiol on experimental IgA nephropathy induced by vomitoxin. Fundam Appl Toxicol. 1995;26(1):107–116. 7657054 10.1006/faat.1995.1080

[272] Yamamoto R, Nagasawa Y, Shoji T, et al. A candidate gene approach to genetic contributors to the development of IgA nephropathy. Nephrol Dial Transplant. 2012;27(3):1020–1030. 21737517 10.1093/ndt/gfr369

[273] Mirfazeli ES, Marashi SA, Kalantari S. In silico prediction of specific pathways that regulate mesangial cell proliferation in IgA nephropathy. Med Hypotheses. 2016;97:38–45. 27876127 10.1016/j.mehy.2016.10.014

[274] Hu S-L, Wang D, Yuan F-L, et al. Identification of key genes and pathways in IgA nephropathy using bioinformatics analysis. Medicine (Baltimore). 2020;99(30):e21372. 32791747 10.1097/MD.0000000000021372 PMC7386957

[275] Yu W, Zhao B, Zhong H, et al. Estrogen receptor alpha expression in renal tissue and its relationship with prognosis in immunoglobulin A nephropathy. Int J Clin Exp Pathol. 2020;13(9):2319–2325. 33042337 PMC7539875

[276] Gross ML. Beneficial effects of estrogens on indices of renal damage in uninephrectomized SHRsp rats. J Am Soc Nephrol. 2004;15(2):348–358. 14747381 10.1097/01.asn.0000105993.63023.d8

[277] Mercantepe T, Unal D, Selli J, et al. Protective effects of estrogen and bortezomib in kidney tissue of post-menopausal rats: an ultrastructural study. Ren Fail. 2016;38(7):1129–1135. 27198138 10.1080/0886022X.2016.1184958

[278] Kasimay O, Sener G, Cakir B, et al. Estrogen protects against oxidative multiorgan damage in rats with chronic renal failure. Ren Fail. 2009;31(8):711–725. 19814639 10.3109/08860220903134563

[279] Maric C, Sandberg K, Hinojosa-Laborde C. Glomerulosclerosis and tubulointerstitial fibrosis are attenuated with 17beta-estradiol in the aging Dahl salt sensitive rat. J Am Soc Nephrol. 2004;15(6):1546–1556. 15153565 10.1097/01.asn.0000128219.65330.ea

[280] Mao S, Xu HUA, Zou L, et al. Estrogen preserves split renal function in a chronic complete unilateral ureteral obstruction animal model. Exp Ther Med. 2014;7(6):1555–1562. 24926343 10.3892/etm.2014.1663 PMC4043623

[281] Kim D, Lee AS, Jung YJ, et al. Tamoxifen ameliorates renal tubulointerstitial fibrosis by modulation of estrogen receptor α-mediated transforming growth factor-β1/Smad signaling pathway. Nephrol Dial Transplant. 2014;29(11):2043–2053. 25031017 10.1093/ndt/gfu240

[282] Dellê H, Rocha JRC, Cavaglieri RC, et al. Antifibrotic effect of tamoxifen in a model of progressive renal disease. J Am Soc Nephrol. 2012;23(1):37–48. 22052053 10.1681/ASN.2011010046 PMC3269918

[283] Diwan V, Small D, Kauter K, et al. Gender differences in adenine-induced chronic kidney disease and cardiovascular complications in rats. Am J Physiol Renal Physiol. 2014;307(11):F1169–F1178. 25209863 10.1152/ajprenal.00676.2013

[284] Ichii O, Nakamura T, Irie T, et al. Close pathological correlations between chronic kidney disease and reproductive organ-associated abnormalities in female cotton rats. Exp Biol Med (Maywood). 2018;243(5):418–427. 29412002 10.1177/1535370218758250 PMC5882030

[285] Moonen L, D'Haese PC, Vervaet BA. Epithelial cell cycle behaviour in the injured kidney. Int J Mol Sci. 2018;19(7): 2038. 10.3390/ijms19072038 PMC6073451 30011818

[286] Kramann R, Kusaba T, Humphreys BD. Who regenerates the kidney tubule? Nephrol Dial Transplant. 2015;30(6):903–910. 25155054 10.1093/ndt/gfu281 PMC4438740

[287] Sánchez DS, Fischer Sigel LK, Azurmendi PJ, et al. Estradiol stimulates cell proliferation via classic estrogen receptor-alpha and G protein-coupled estrogen receptor-1 in human renal tubular epithelial cell primary cultures. Biochem Biophys Res Commun. 2019;512(2):170–175. 30879772 10.1016/j.bbrc.2019.03.056

[288] Sun J, Langer WJ, Devish K, et al. Compensatory kidney growth in estrogen receptor-alpha null mice. Am J Physiol Renal Physiol. 2006;290(2):F319–F323. 16159896 10.1152/ajprenal.00271.2005

[289] Tieu A, House AA, Urquhart BL. Drug disposition issues in CKD: implications for drug discovery and regulatory approval. Adv Chronic Kidney Dis. 2016;23(2):63–66. 26979144 10.1053/j.ackd.2016.01.013

[290] Lin H-F, Liao K-F, Chang C-M, et al. Correlation of the tamoxifen use with the increased risk of deep vein thrombosis and pulmonary embolism in elderly women with breast cancer: a case-control study. Medicine (Baltimore). 2018;97(51):e12842. 30572423 10.1097/MD.0000000000012842 PMC6320050

[291] Adomaityte J, Farooq M, Qayyum R. Effect of raloxifene therapy on venous thromboembolism in postmenopausal women. A meta-analysis. Thromb Haemost. 2008;99(02):338–342. 18278183

[292] Isakova T, Nickolas TL, Denburg M, et al. KDOQI US commentary on the 2017 KDIGO Clinical Practice Guideline update for the diagnosis, evaluation, prevention, and treatment of Chronic Kidney Disease-Mineral and Bone Disorder (CKD-MBD). Am J Kidney Dis. 2017;70(6):737–751. 28941764 10.1053/j.ajkd.2017.07.019

[293] Chen H, Han X, Cui Y, et al. Parathyroid hormone fragments: new targets for the diagnosis and treatment of chronic kidney disease-mineral and bone disorder. Biomed Res Int. 2018;2018:9619253. 30627584 10.1155/2018/9619253 PMC6304519

[294] Webster R, Sheriff S, Faroqui R, et al. Klotho/fibroblast growth factor 23- and PTH-independent estrogen receptor-α-mediated direct downregulation of NaPi-IIa by estrogen in the mouse kidney. Am J Physiol Renal Physiol. 2016;311(2):F249–F259. 27194721 10.1152/ajprenal.00542.2015 PMC5008677

[295] Meng J, Ohlsson C, Laughlin GA, Osteoporotic Fractures in Men (MrOs) Study Group, et al. Associations of estradiol and testosterone with serum phosphorus in older men: the Osteoporotic Fractures in Men study. Kidney Int. 2010;78(4):415–422. 20531458 10.1038/ki.2010.161 PMC3787687

[296] Ix JH, Chonchol M, Laughlin GA, et al. Relation of sex and estrogen therapy to serum fibroblast growth factor 23, serum phosphorus, and urine phosphorus: the Heart and Soul Study. Am J Kidney Dis. 2011;58(5):737–745. 21855188 10.1053/j.ajkd.2011.06.011 PMC3199317

[297] Masaki H, Imanishi Y, Naka H, et al. Bazedoxifene improves renal function and increases renal phosphate excretion in patients with postmenopausal osteoporosis. J Bone Miner Metab. 2020;38(3):405–411. 31897746 10.1007/s00774-019-01073-1

[298] Suzuki H, Kondo K. Chronic kidney disease in postmenopausal women. Hypertens Res. 2012;35(2):142–147. 21900940 10.1038/hr.2011.155

[299] Khairallah P, Nickolas TL. Management of osteoporosis in CKD. Clin J Am Soc Nephrol. 2018;13(6):962–969. 29487093 10.2215/CJN.11031017 PMC5989687

[300] Haghverdi F, Farbodara T, Mortaji S, et al. Effect of raloxifene on parathyroid hormone in osteopenic and osteoporotic postmenopausal women with chronic kidney disease stage 5. Iran J Kidney Dis. 2014;8(6):461–466. 25362221

[301] Saito O, Saito T, Asakura S, et al. The effects of raloxifene on bone turnover markers and bone mineral density in women on maintenance hemodialysis. Clin Exp Nephrol. 2011;15(1):126–131. 21069410 10.1007/s10157-010-0366-0

[302] Nagatoya K, Nishimoto K, Shibahara N, Hokusetsu Renal Osteodystrophy Study Group, et al. Effects of raloxifene on bone metabolism in postmenopausal women on chronic hemodialysis. Clin Exp Nephrol. 2015;19(5):939–946. 25504368 10.1007/s10157-014-1065-z

[303] Ishani A, Blackwell T, Jamal SA, et al. The effect of raloxifene treatment in postmenopausal women with CKD. JASN. 2008;19(7):1430–1438. 18400939 10.1681/ASN.2007050555 PMC2440292

[304] Heilberg IP, Hernandez E, Alonzo E, et al. Estrogen receptor (ER) gene polymorphism may predict the bone mineral density response to raloxifene in postmenopausal women on chronic hemodialysis. LRNF. 2005;27(2):155–161. 15807179

[305] Zhang S, Guo Y, Zou H, et al. Effect of estrogen deficiency on the fixation of titanium implants in chronic kidney disease mice. Osteoporos Int. 2015;26(3):1073–1080. 25366374 10.1007/s00198-014-2952-6

[306] Guo Y, Sun N, Duan X, et al. Estrogen deficiency leads to further bone loss in the mandible of CKD mice. PLoS One. 2016;11(2):e0148804. 26886008 10.1371/journal.pone.0148804 PMC4757532

[307] Newman CL, Creecy A, Granke M, et al. Raloxifene improves skeletal properties in an animal model of cystic chronic kidney disease. Kidney Int. 2016;89(1):95–104. 26489025 10.1038/ki.2015.315 PMC4840093

[308] Kanatani M, Sugimoto T, Takahashi Y, et al. Estrogen via the estrogen receptor blocks cAMP-mediated parathyroid hormone (PTH)-stimulated osteoclast formation. J Bone Miner Res. 1998;13(5):854–862. 9610750 10.1359/jbmr.1998.13.5.854

[309] Liu B-Y, Wu P-W, Bringhurst FR, et al. Estrogen inhibition of PTH-stimulated osteoclast formation and attachment in vitro: involvement of both PKA and PKC. Endocrinology. 2002;143(2):627–635. 11796519 10.1210/endo.143.2.8614

[310] Romagnani P, Remuzzi G, Glassock R, et al. Chronic kidney disease. Nat Rev Dis Primers. 2017;3:17088. 29168475 10.1038/nrdp.2017.88

[311] Voelkl J, Lang F, Eckardt K-U, et al. Signaling pathways involved in vascular smooth muscle cell calcification during hyperphosphatemia. Cell Mol Life Sci. 2019;76(11):2077–2091. 30887097 10.1007/s00018-019-03054-z PMC6502780

[312] Rzewuska-Lech E, Jayachandran M, Fitzpatrick LA, et al. Differential effects of 17beta-estradiol and raloxifene on VSMC phenotype and expression of osteoblast-associated proteins. Am J Physiol Endocrinol Metab. 2005;289(1):E105–E112. 15713688 10.1152/ajpendo.00366.2004

[313] Nanao-Hamai M, Son B-K, Hashizume T, et al. Protective effects of estrogen against vascular calcification via estrogen receptor α-dependent growth arrest-specific gene 6 transactivation. Biochem Biophys Res Commun. 2016;480(3):429–435. 27771246 10.1016/j.bbrc.2016.10.066

[314] Kim SA, Lee KY, Kim J-R, et al. Estrogenic compound attenuates angiotensin II-induced vascular smooth muscle cell proliferation through interaction between LKB1 and estrogen receptor α. J Pharmacol Sci. 2016;132(1):78–85. 27665370 10.1016/j.jphs.2016.09.001

[315] Chang SP, Yang WS, Lee KS, et al. Effects of hormonal replacement therapy on oxidative stress and total antioxidant capacity in postmenopausal hemodialysis patients. Ren Fail. 2002;24(1):49–57. 11921698 10.1081/jdi-120002660

[316] Kendrick J, Chonchol M. Novel therapeutic options for the treatment of mineral metabolism abnormalities in end stage renal disease. Semin Dial. 2015;28(6):610–619. 26278462 10.1111/sdi.12412 PMC4626263

[317] National Kidney Foundation. K/DOQI clinical practice guidelines for bone metabolism and disease in chronic kidney disease. Am J Kidney Dis. 2003;42(4 Suppl 3):S1–S201. 14520607

[318] Ma H, Ouyang C, Huang Y, et al. Comparison of microwave ablation treatments in patients with renal secondary and primary hyperparathyroidism. Ren Fail. 2020;42(1):66–76. 31928297 10.1080/0886022X.2019.1707097 PMC7006805

[319] Yu M-A, Yao L, Zhang L, et al. Safety and efficiency of microwave ablation for recurrent and persistent secondary hyperparathyroidism after parathyroidectomy: a retrospective pilot study. Int J Hyperthermia. 2016;32(2):180–186. 26606889 10.3109/02656736.2015.1101788

[320] Lin X-H, Lin C-C, Wang Y-J, et al. Risk factors of the peptic ulcer bleeding in aging uremia patients under regular hemodialysis. J Chin Med Assoc. 2018;81(12):1027–1032. 29778548 10.1016/j.jcma.2018.03.007

[321] Wakasugi M, Matsuo K, Kazama JJ, et al. Higher mortality due to intracerebral hemorrhage in dialysis patients: a comparison with the general population in Japan. Ther Apher Dial. 2015;19(1):45–49. 25196294 10.1111/1744-9987.12192

[322] Sohal AS, Gangji AS, Crowther MA, et al. Uremic bleeding: pathophysiology and clinical risk factors. Thromb Res. 2006;118(3):417–422. 15993929 10.1016/j.thromres.2005.03.032

[323] Hunt BJ. Bleeding and coagulopathies in critical care. N Engl J Med. 2014;370(9):847–859. 24571757 10.1056/NEJMra1208626

[324] Hussain S, Siddiqui AN, Baxi H, et al. Warfarin use increases bleeding risk in hemodialysis patients with atrial fibrillation: a meta-analysis of cohort studies. J Gastroenterol Hepatol. 2019;34(6):975–984. 30614083 10.1111/jgh.14601

[325] Ishii M, Ogawa H, Unoki T, et al. Relationship of hypertension and systolic blood pressure with the risk of stroke or bleeding in patients with atrial fibrillation: the Fushimi AF Registry. Am J Hypertens. 2017;30(11):1073–1082. 28575205 10.1093/ajh/hpx094

[326] Yamashita Y, Takagi D, Hamatani Y, et al. Clinical characteristics and outcomes of dialysis patients with atrial fibrillation: the Fushimi AF Registry. Heart Vessels. 2016;31(12):2025–2034. 26973346 10.1007/s00380-016-0818-x

[327] Gonzalez J, Bryant S, Hermes-DeSantis ER. Transdermal estradiol for the management of refractory uremic bleeding. Am J Health Syst Pharm. 2018;75(9):e177–e183. 29691259 10.2146/ajhp170241

[328] Pei J, Harakalova M, den Ruijter H, et al. Cardiorenal disease connection during post-menopause: the protective role of estrogen in uremic toxins induced microvascular dysfunction. Int J Cardiol. 2017;238:22–30. 28341374 10.1016/j.ijcard.2017.03.050

[329] Hedges SJ, Dehoney SB, Hooper JS, et al. Evidence-based treatment recommendations for uremic bleeding. Nat Clin Pract Nephrol. 2007;3(3):138–153. 17322926 10.1038/ncpneph0421

[330] Szilagyi A, Ghali MP. Pharmacological therapy of vascular malformations of the gastrointestinal tract. Can J Gastroenterol. 2006;20(3):171–178. 16550261 10.1155/2006/859435 PMC2582970

[331] Muftah M, Mulki R, Dhere T, et al. Diagnostic and therapeutic considerations for obscure gastrointestinal bleeding in patients with chronic kidney disease. Ann Gastroenterol. 2019;32(2):113–123. 30837783 10.20524/aog.2018.0341 PMC6394262

[332] Mosconi G, Mambelli E, Zanchelli F, et al. Severe gastrointestinal bleeding in a uremic patient treated with estrogen-progesterone therapy. Int J Artif Organs. 1999;22(5):313–316. 10467929

[333] Hermans C, Goffin E, Horsmans Y, et al. Watermelon stomach. An unusual cause of recurrent upper GI tract bleeding in the uraemic patient: efficient treatment with oestrogen-progesterone therapy. Nephrol Dial Transplant. 1996;11(5):871–874. 8671914 10.1093/oxfordjournals.ndt.a027418

[334] Bali A, Hix JK, Kouides P. Safe and effective use of chronic transdermal estradiol for life-threatening uremic bleeding in a patient with coronary artery disease. Nephron Extra. 2014;4(2):134–137. 25337082 10.1159/000365480 PMC4164069

[335] Viganò G, Zoja C, Corna D, et al. 17 beta-estradiol is the most active component of the conjugated estrogen mixture active on uremic bleeding by a receptor mechanism. J Pharmacol Exp Ther. 1990;252(1):344–348. 2153805

[336] Virdis A, Ghiadoni L, Pinto S, et al. Mechanisms responsible for endothelial dysfunction associated with acute estrogen deprivation in normotensive women. Circulation. 2000;101(19):2258–2263. 10811592 10.1161/01.cir.101.19.2258

[337] Brunini TMC, da Silva CD, Siqueira MAS, et al. Uremia, atherothrombosis and malnutrition: the role of L-arginine-nitric oxide pathway. Cardiovasc Hematol Disord Drug Targets. 2006;6(2):133–140. 16787198 10.2174/187152906777441821

[338] Brunini TMC, Mendes-Ribeiro AC, Ellory JC, et al. Platelet nitric oxide synthesis in uremia and malnutrition: a role for L-arginine supplementation in vascular protection? Cardiovasc Res. 2007;73(2):359–367. 17078937 10.1016/j.cardiores.2006.09.019

[339] Gurney EP, Nachtigall MJ, Nachtigall LE, et al. The Women's Health Initiative trial and related studies: 10 years later: a clinician's view. J Steroid Biochem Mol Biol. 2014;142:4–11. 24172877 10.1016/j.jsbmb.2013.10.009

[340] Rossouw JE, Manson JE, Kaunitz AM, et al. Lessons learned from the Women's Health Initiative trials of menopausal hormone therapy. Obstet Gynecol. 2013;121(1):172–176. 23262943 10.1097/aog.0b013e31827a08c8 PMC3547645

[341] K/DOQI Workgroup. K/DOQI clinical practice guidelines for cardiovascular disease in dialysis patients. Am J Kidney Dis. 2005;45(4 Suppl 3):S1–S153. 15806502

[342] Kramer HM, Curhan GC, Singh A, Hemodialysis and Estrogen Levels in Postmenopausal Patients Study Group. Permanent cessation of menses and postmenopausal hormone use in dialysis-dependent women: the HELP study. Am J Kidney Dis. 2003;41(3):643–650. 12612988 10.1053/ajkd.2003.50126

[343] Vellanki K, Hou S. Menopause in CKD. Am J Kidney Dis. 2018;71(5):710–719. 29530509 10.1053/j.ajkd.2017.12.019

[344] Dobrowolski C, Clark EG, Sood MM. Venous thromboembolism in chronic kidney disease: epidemiology, the role of proteinuria, CKD severity and therapeutics. J Thromb Thrombolysis. 2017;43(2):241–247. 27738784 10.1007/s11239-016-1437-1

[345] Lu HY, Liao KM. Increased risk of deep vein thrombosis in end-stage renal disease patients. BMC Nephrol. 2018;19(1):204. 30115029 10.1186/s12882-018-0989-z PMC6097196

[346] Huang M-J, Wei R-b, Wang Y, et al. Blood coagulation system in patients with chronic kidney disease: a prospective observational study. BMJ Open. 2017;7(5):e014294. 10.1136/bmjopen-2016-014294 PMC5541338 28576889

[347] Shashar M, Belghasem ME, Matsuura S, et al. Targeting STUB1-tissue factor axis normalizes hyperthrombotic uremic phenotype without increasing bleeding risk. Sci Transl Med. 2017;9(417): eaam8475. 10.1126/scitranslmed.aam8475 PMC5854487 29167396

[348] DeLoughery TG. Estrogen and thrombosis: controversies and common sense. Rev Endocr Metab Disord. 2011;12(2):77–84. 21559819 10.1007/s11154-011-9178-0

[349] Ahmed SB, Ramesh S. Sex hormones in women with kidney disease. Nephrol Dial Transplant. 2016;31(11):1787–1795. 27190328 10.1093/ndt/gfw084

[350] Andreoli SP. Hormone replacement therapy in postmenopausal women with end-stage renal disease (1). Kidney Int. 2000;57(1):341–342. 10620218 10.1046/j.1523-1755.2000.00850.x

[351] Hoshi-Fukushima R, Nakamoto H, Imai H, et al. Estrogen and angiotensin II interactions determine cardio-renal damage in Dahl salt-sensitive rats with heart failure. Am J Nephrol. 2008;28(3):413–423. 18097133 10.1159/000112806

